# Managing Experimental 3D Structures in the Beyond‐Rule‐of‐5 Chemical Space: The Case of Rifampicin

**DOI:** 10.1002/chem.202100961

**Published:** 2021-06-10

**Authors:** Giuseppe Ermondi, Francesca Lavore, Maura Vallaro, Guido Tiana, Francesca Vasile, Giulia Caron

**Affiliations:** ^1^ Molecular Biotechnology and Health Sciences Dept. Università degli Studi di Torino via Quarello 15 10135 Torino Italy; ^2^ Department of Chemistry Università degli Studi di Milano via Golgi 19 20133 Milano Italy; ^3^ Department of Physics and Center for Complexity and Biosystems Università degli Studi di Milano and INFN via Celoria 16 20133 Milano Italy

**Keywords:** bRo5, chameleonicity, intramolecular hydrogen bonds, macrocycles, NMR spectroscopy

## Abstract

The beyond‐Rule‐of‐5 (bRo5) chemical space is a source of new oral drugs and includes large and flexible compounds. Because of their size and conformational variability, bRo5 molecules assume different privileged conformations in the compartments of human body, i. e., they can exhibit chameleonic properties. The elucidation of the ensemble of 3D structures explored by such molecules under different conditions is therefore critical to check the role played by chameleonicity to modulate cell permeability. Here we characterized the conformational ensembles of rifampicin, a bRo5 drug, in polar and nonpolar solvents and in the solid state. We performed NMR experiments, analyzed their results with a novel algorithm and set‐up a pool of *ad hoc in silico* strategies to investigate crystallographic structures retrieved from the CSD. Moreover, a polarity descriptor often related to permeability (SA‐3D‐PSA) was calculated for all the conformers and its variation with the environment analyzed. Results showed that the conformational behavior of rifampicin in solution and in the solid state is not superposable. The identification of dynamic intramolecular hydrogen bonds can be assessed by NMR spectroscopy but not by X‐ray structures. Moreover, SA‐3D‐PSA revealed that dynamic IMHBs do not provide rifampicin with chameleonic properties. Overall, this study highlights that the peculiarity of rifampicin, which is cell permeable probably because of the presence of static IMHBs but is devoid of any chameleonic behavior, can be assessed by a proper analysis of experimental 3D structures.

## Graphical abstract

**The bRo5 space** includes large and flexible drug candidates which may assume different privileged conformations and exhibit chameleonic properties. We characterized the conformational ensembles of rifampicin in polar and nonpolar solvents and in the solid state. We performed NMR experiments, analyzed CSD structures and calculated a polarity descriptor. Results show that rifampicin is not a chameleon and its conformational behavior in solution and in the solid state is not superposable.

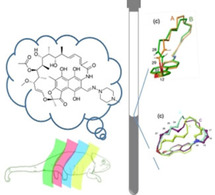

## Introduction

A dramatic increase in the number of drugs approved in the chemical space outside of Lipinski's rule of 5, i. e. the so called beyond rule of 5 (bRo5) space, has been registered in the last years.^[1]^ This is due to the evidence that large and flexible compounds may modulate difficult‐to‐drug targets playing a crucial role in major unmet diseases like cancer and neurodegenerative diseases.^[2]^

Because of their size and conformational variability, bRo5 molecules can assume different privileged conformations in the various compartments of the human body. Some authors^[3]^ recently outlined how this capacity of compounds to adapt to different environments (i. e. ‘chameleonicity’ skills) is crucial to rationalize the good permeability properties exhibited by flexible molecules and more recently chameleonicity has been shown to be a molecular property that deserves being incorporated in the design of new oral drugs in the bRo5 chemical space.[^4^, ^5^] Therefore, unlike small molecules, bRo5 compounds cannot be represented *a priori* as a single average conformation but rather a pool of biorelevant conformations should be individuated as early as possible in any bRo5 drug discovery program.

Recent studies showed that up to now, computational tools have significant issues in the identification of biorelevant conformations of large and flexible structures.[^3^, ^6^] For example, the design of new oral drugs calls for computational tools able to predict *in vitro* properties like solubility and permeability.^[5]^ In a recent study on a set of bRo5 drugs,^[6]^ some of us showed how a robust model relating linearly passive cell permeability with the minimum solvent accessible 3D polar surface area calculated on crystallographic structures became significantly weaker when computed structures replaced experimental ones. This and other examples clearly outline that there is the need of experimental 3D structures to make more efficient the drug discovery process.

The determination of the experimental structures of large and flexible molecules is far from trivial. X‐ray crystallography is largely used since a lot of information can be extracted from X‐ray online databases using tailored instruments. For example, the Cambridge Structural Database (CSD) can be explored with tools implemented in the Mercury software.^[7]^ Limits of X‐ray data are also well known. For instance, only a limited pool of conformations is available because of the crystal environment.

Nuclear magnetic resonance (NMR) returns signals that reflect the average conformation of the molecule in solution, signals that must be interpreted properly by computational models to describe the conformations accessible to the molecule. A common approach to deconvolute molecular conformers from NMR spectra is the NAMFIS algorithm.^[8]^ Recently, a method that combines NMR data with molecular dynamics (MD) simulations, within the theoretical framework of the principle of maximum entropy, has proven useful to reproduce ensembles of molecular conformations with the correct statistical weights.[^9^, ^10^, ^11^] The basic idea was to sample the conformations of the flexible molecule by MD with a standard force field that covers a priori knowledge of the molecular interactions, corrected iteratively to match the experimental average data.

**Figure 1:**
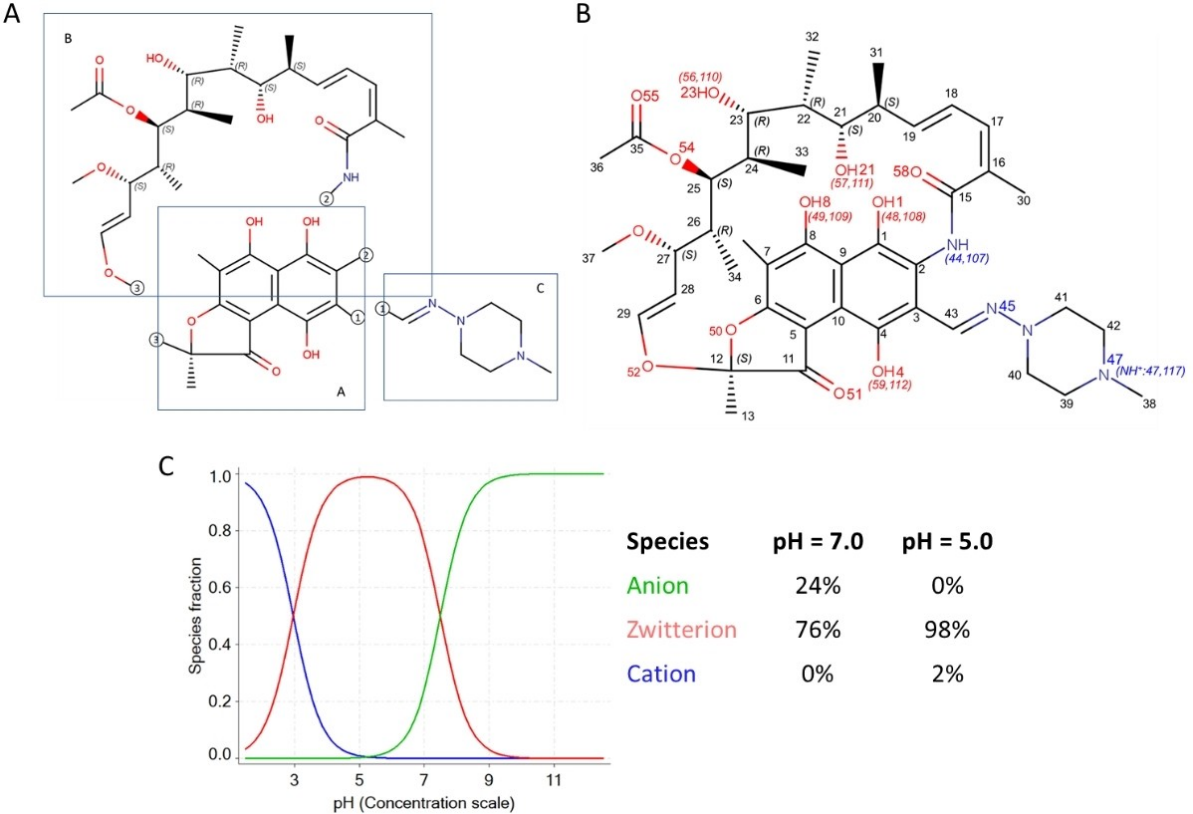
Rifampicin structure, numbering and ionization properties. A) the three main moieties: the naphthohydroquinone system fused with the furanone ring; the flexible chain (called ansa); the extra‐cycle moiety formed by a (4‐methyl‐1‐piperazinyl)‐iminomethyl group; B) atomic numbering; and C) aqueous ionization profile.

Rifampicin is a bRo5 compound and belongs to a wide group of macrocyclic antibiotics called rifamycins that have been at the top of clinically used pharmaceuticals against tuberculosis over the last 35 years. It binds to bacterial DNA‐dependent RNA polymerase and prevents RNA synthesis via occlusion of the elongating RNA strand.^[1]^ The macrocycle includes two distinct moieties (Figure 1): a rigid section formed by a naphthohydroquinone system fused with a furanone ring and a flexible chain (called ansa) partially blocked by cycle restrain. Moreover, it has extra‐cycle moiety formed by a (4‐methyl‐1‐piperazinyl)‐iminomethyl group (see Figure 1). Potentiometric measurements provided two p*K*_a_ values for rifampicin: 2.97 (acidic) and 7.50 (basic).^[5]^ The ionization profile in water is reported in Figure 1C. Although predominantly zwitterionic in a large pH range, rifampicin isoelectric point is 5.2 and thus at physiological pH the anionic species is also significatively present in solution (about 44 % at pH=7.4).

Rifampicin cell permeability in a Caco2 system is about 1×10^−6^ cm/s with a significant value of the efflux ratio (ER=14.4).^[5]^ Overall rifampicin is a macrocyclic bRo5 compound showing good permeability properties.

In this study we determined the NMR conformational ensembles of rifampicin in polar and nonpolar environment using a method that provides a realistic ensemble of conformers and compared the results with crystallographic structures retrieved from CSD. In particular, we focused on the intriguing intramolecular hydrogen bond (IMHB) network exhibited by the macrocycle also in relation with its ionization state recently experimentally characterized in nonpolar media.^[12]^ To relate structural features with molecular properties, we calculated the polar surface area (SA‐3D‐PSA, a major determinant of cell permeability) for all the experimental conformations. From the analysis of SA‐3D‐PSA variation with the environment, in view of the permeability of rifampicin, we explored its potential chameleonicity.

Overall, this study highlights the different information content of X‐ray and NMR conformational ensembles, provides indications about the use of experimental structures in bRo5 drug discovery and suggests structural hypothesis to rationalize the favorable permeability properties exhibited by the drug.

## Results AND Discussion

### Preliminary analysis

A pool of three common 2D molecular descriptors (the Kier flexibility index, PHI, the number of hydrogen bond donor, HBD, and acceptor, HBA, atoms) were calculated (Table 1) to compare rifampicin with three macrocyclic bRo5 drugs for which the presence of IMHBs has been associated to permeability properties: cyclosporin A (CsA),^[13]^ erythromycin and roxithromycin.^[14]^ Calculated values are reported in Table 1. Notably, rifampicin and erythromycin show a similar flexibility, lower than that of roxithromycin and very much lower than that of CsA. Rifampicin has 6 HBD groups, whereas the three remaining molecules have 5 and an intermediate value of HBA groups.

**Table 1:** 2D molecular descriptors: PHI (flexibility indicator), HBD (number of hydrogen bond donor groups) and HBA (number of hydrogen bond acceptors groups). HBA and HBD are limited to N and O atoms.

Compound	PHI	HBD	HBA
Cyclosporin A	33.6	5	25
Erythromycin	14.9	5	14
Roxithromycin	19.1	5	17
Rifampicin	14.8	6	16

As pointed out elsewhere, a number of HBD and HBA groups greater than zero and a certain amount of flexibility are mandatory conditions to allow the formation of IMHB (geometrical restrictions should be respected too).^[15]^ Therefore, Table 1 suggests that at least in theory rifampicin can form IMHBs.

**Figure 2:**
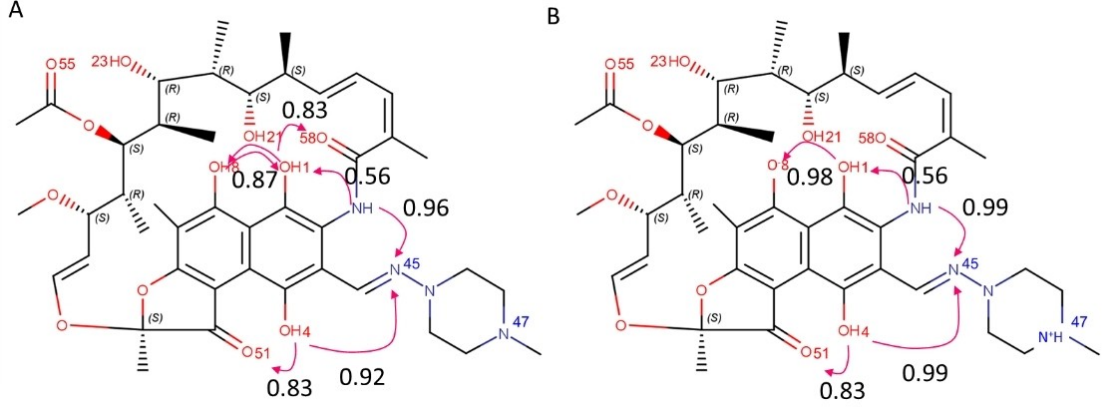
Rifampicin HBP propensity calculated by Mercury: A) neutral and B) zwitterionic form.

To obtain a quantitative evaluation of the propensity of rifampicin to form IMHBs, we submitted an *in silico* structure of neutral and zwitterionic rifampicin to Mercury (calculations do not depend on the selected conformation, see Methods).^[16]^ This tool computes a probability score (range: 0‐1), named HB propensity (HBP), for the formation of a hydrogen bond between a specific donor and acceptor atom. HBP is a knowledge‐based function obtained by a statistical analysis of the HBs information extracted by the structures collected in the Cambridge Structural Databases (CSD). Results are in Figure 2 and show that rifampicin is expected to easily form IMHBs in both ionization states.

### Crystallographic structures

#### Structure selection

Crystallographic databases are the main source of 3D structures for compounds of pharmaceutical interest. In this study, 21 entries of rifampicin were retrieved in the CSD database (version 5.41, update Aug 2020) and analyzed for their quality (Table S1). Despite advances in the field of powder diffraction resolution, we preferred to focus on structures obtained with single‐crystal techniques due to better accuracy in atom positions.^[17]^ Therefore, we discarded powder structure, i. e. LOPZEX and LOPZEX10. HAXWUA and RIFAMP are both referred to rifampicin pentahydrate. Here we considered only HAXWUA since it was redetermined with the correct ionization state, i. e. zwitterionic.^[18]^ Similarly OWELAS and OWELUY are referred to hydrate forms of rifampicin but are neutral. Since very small amount of water are sufficient to guarantee the presence of the zwitterionic form[^12^, ^18^] these two structures were discarded. Finally, 16 CSD entries were retained. Notably, only in MAPHES rifampicin is in the neutral state whereas all remaining entries concern the zwitterionic species. This is related to the evidence that most compounds were crystallized from water or organic solvents containing mobile hydrogen atoms that enable the zwitterion formation.^[19]^ Finally, hydrogen positions were normalized, even if we verified that the IMHBs network is not influenced by the hydrogen normalization.

#### Cluster identification

**Figure 3:**
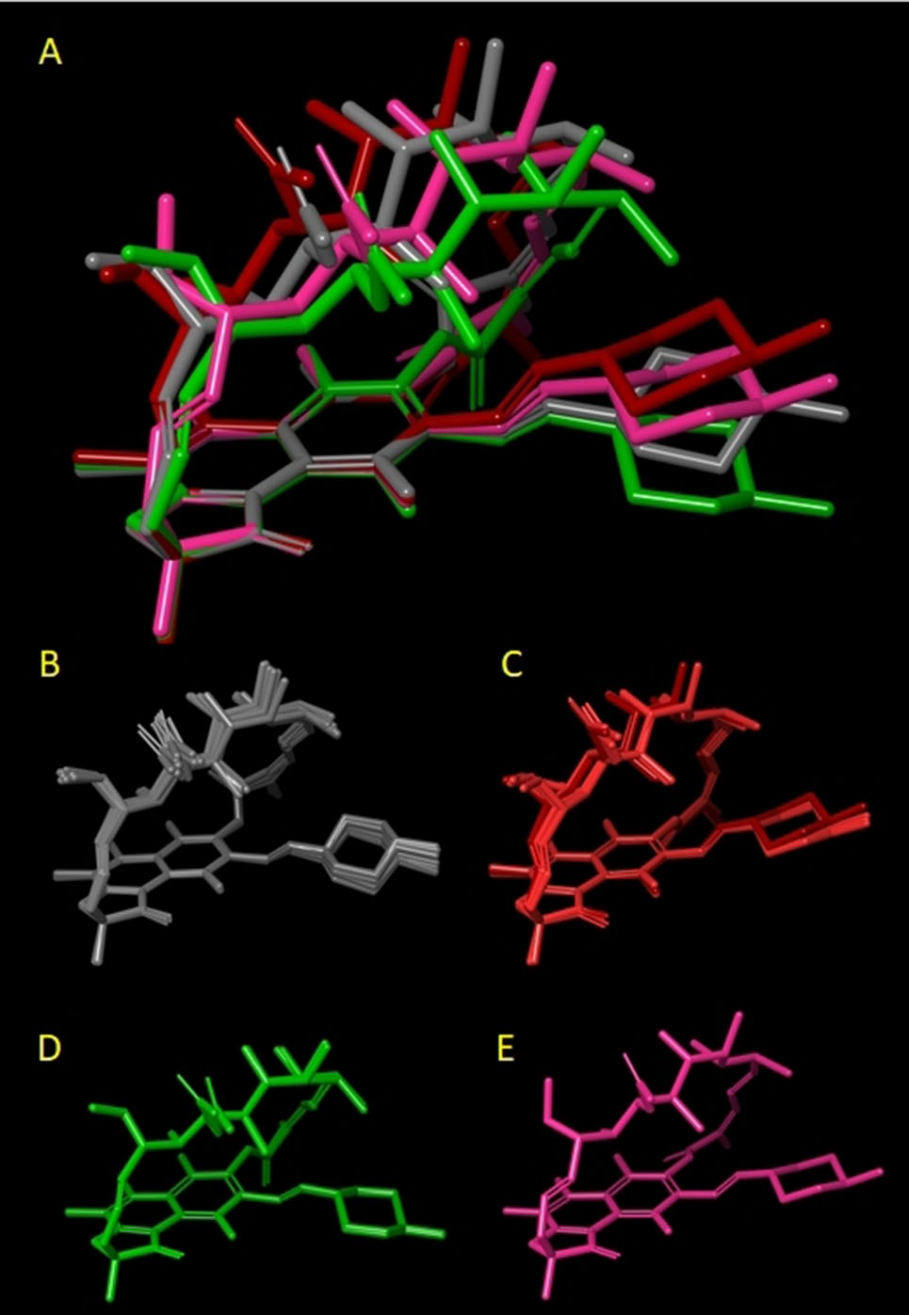
Superposition of the X‐ray structures using the shared naphthohydroquinone moiety as a template, the clusters are also shown separately in the same orientation to gain clarity and are colored according to Table 2; A) all structures; B) cluster with YELYAS, YELYIA, YELYOG, YELZAT, YELZEX, YELZIB, YELZOH, YELZUN, YEMBAW, in grey; C) cluster with HAXWUA, MAPHIW, YELYUM, YEMCIF, in red; D) cluster in with YELXUL and YELYEW, in green; E) MAPHES.

The 16 X‐ray structures were aligned using the naphthohydroquinone moiety as a template (Figure 3). RMSD values are in Table 2. An RMSD value equal to 0.5 was considered a convenient threshold to assign two structures to the same cluster. Four clusters were obtained:

**Table 2:** RMSD values calculated superposing the 16 X‐ray structures of rifampicin using all heavy atoms. The four clusters are in different colors.

	HAXWUA	MAPHES	MAPHIW	YELXUL	YELYAS	YELYEW	YELYIA	YELYOG	YELYUM	YELZAT	YELZEX	YELZIB	YELZOH	YELZUN	YEMBAW	YEMCIF
HAXWUA	0.000	0.619	0.132	0.801	0.785	0.793	0.759	0.829	0.165	0.763	0.749	0.897	0.712	0.736	0.711	0.212
MAPHES	0.619	0.000	0.685	0.640	0.720	0.660	0.664	0.731	0.512	0.654	0.609	0.751	0.630	0.661	0.605	0.566
MAPHIW	0.132	0.685	0.000	0.799	0.759	0.785	0.744	0.807	0.235	0.754	0.749	0.880	0.704	0.722	0.711	0.266
YELXUL	0.801	0.640	0.799	0.000	0.621	0.179	0.604	0.596	0.691	0.604	0.614	0.594	0.602	0.614	0.618	0.759
YELYAS	0.785	0.720	0.759	0.621	0.000	0.618	0.154	0.162	0.707	0.191	0.247	0.282	0.188	0.174	0.223	0.749
YELYEW	0.793	0.660	0.785	0.179	0.618	0.000	0.582	0.592	0.682	0.621	0.627	0.561	0.612	0.603	0.639	0.748
YELYIA	0.759	0.664	0.744	0.604	0.154	0.582	0.000	0.157	0.670	0.214	0.217	0.247	0.176	0.128	0.216	0.718
YELYOG	0.829	0.731	0.807	0.596	0.162	0.592	0.157	0.000	0.746	0.204	0.238	0.203	0.195	0.165	0.264	0.804
YELYUM	0.165	0.512	0.235	0.691	0.707	0.682	0.670	0.746	0.000	0.687	0.665	0.804	0.635	0.659	0.629	0.156
YELZAT	0.763	0.654	0.754	0.604	0.191	0.621	0.214	0.204	0.687	0.000	0.113	0.300	0.122	0.150	0.141	0.745
YELZEX	0.749	0.609	0.749	0.614	0.247	0.627	0.217	0.238	0.665	0.113	0.000	0.315	0.127	0.151	0.126	0.723
YELZIB	0.897	0.751	0.880	0.594	0.282	0.561	0.247	0.203	0.804	0.300	0.315	0.000	0.307	0.261	0.366	0.867
YELZOH	0.712	0.630	0.704	0.602	0.188	0.612	0.176	0.195	0.635	0.122	0.127	0.307	0.000	0.109	0.139	0.696
YELZUN	0.736	0.661	0.722	0.614	0.174	0.603	0.128	0.165	0.659	0.150	0.151	0.261	0.109	0.000	0.183	0.715
YEMBAW	0.711	0.605	0.711	0.618	0.223	0.639	0.216	0.264	0.629	0.141	0.126	0.366	0.139	0.183	0.000	0.673
YEMCIF	0.212	0.566	0.266	0.759	0.749	0.748	0.718	0.804	0.156	0.745	0.723	0.867	0.696	0.715	0.673	0.000

- the most populated cluster which contains 9 structures (in grey in Table 2 and Figure 3): YELYAS, YELYIA, YELYOG, YELZAT, YELZEX, YELZIB, YELZOH, YELZUN, YEMBAW;
- a second cluster with four structures (in red in Table 2 and Figure 3): HAXWUA, MAPHIW, YELYUM, YEMCIF;
- a third cluster (in green in in Table 2 and Figure 3): YELXUL and YELYEW;
- the unique neutral structure that does not fit to any cluster (in magenta in Table 2 and Figure 3): MAPHES

A visual inspection of the structures (Figure 3) reveals that clusters mostly differ in the reciprocal orientation of the two main moieties, i. e., the ansa and the (4‐methyl‐1‐piperazinyl)‐iminomethyl groups.

#### Conformational variability

**Figure 4:**
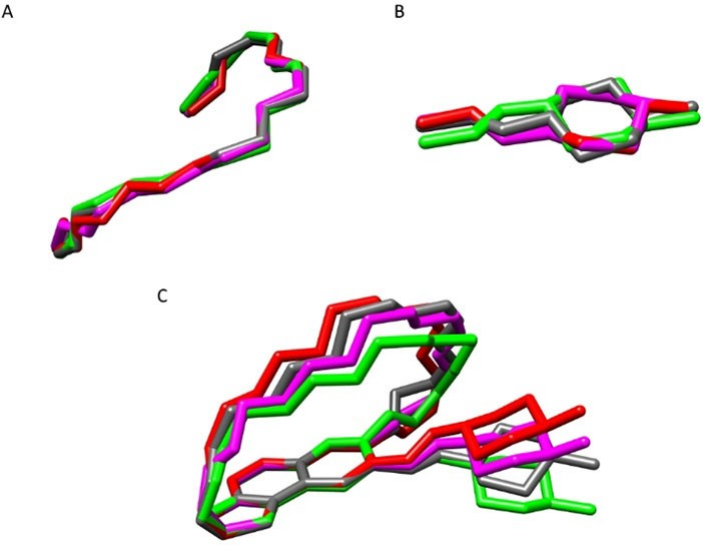
Superposition of the representative structures of the four clusters (HAXWUA in red, MAPHES in magenta, YELXUL in green and YELZOH in grey). using different section of the molecules: A) the ansa; B) the (4‐methyl‐1‐piperazinyl)‐iminomethyl group; C) the naphthohydroquinone system.

To investigate the contribution of the different moieties to conformational variability, we selected one representative structure from each cluster (see above). Then we performed three alignment runs by superposing a) the ansa atoms, b) the (4‐methyl‐1‐piperazinyl)‐iminomethyl group, and c) the naphthohydroquinone system. The superposition of the ansa atoms (Figure 4A) reveals that the conformation variability of this region is poor despite the different orientation of the amide group in the YELZOH cluster (grey atoms in Figure 4A). The (4‐methyl‐1‐piperazinyl)‐iminomethyl group shows a somewhat greater flexibility than the ansa moiety mostly due to the different orientations of the imine moiety in the HAXWUA cluster (in green Figure 4B). Furthermore, the piperazinyl ring in the YELZOH cluster assumes a different chair conformations respect to the others. Finally, we superposed the four rifampicin structures using the rigid naphthohydroquinone system (Figure 4C). This last superposition shows that the ansa moiety assumes a few different orientations due to rigid movements and the same is basically true also for the (4‐methyl‐1‐piperazinyl)‐iminomethyl groups.

Flexibility of rifampicin in the solid state was also explored through the analysis of the thermal ellipsoids (Figure S1, for YELYUM and YEMCIF data are not available probably due to a low resolution). The thermal ellipsoids are related to the magnitude and directions of the thermal vibration of atoms in crystals and thus give a rough estimation of the flexibility of the different portions of the molecule. Basically, we can assume that the ring atoms in the naphthohydroquinone ring assume well‐defined positions (smaller thermal ellipsoids) whereas terminal atoms such as oxygens in hydroxyl groups located in the ansa moiety are expected to exhibit larger flexibility (larger thermal ellipsoids). Figure S1 shows that the thermal ellipsoids of the ansa moiety are comparable to those due to the naphthohydroquinone group. This evidence confirms the poor conformational variability of both moieties. Analogous conclusions can be drawn for the (4‐methyl‐1‐piperazinyl)‐iminomethyl group although the dimensions of the thermal ellipsoids of HAXWUA suggested a slightly greater flexibility.

Summing up, the overall conformational flexibility of rifampicin is mostly due to the different orientations of the ansa region in relation to the naphthohydroquinone moiety and not to the flexibility of the ansa itself.

#### IMHB network

Intramolecular interactions are the major determinants of chameleonicity^[5]^ and IMHBs are the most important among them. IMHBs were recently classified into static and dynamic.^[15]^ Static IMHBs are always formed whereas dynamic IMHBs are formed in nonpolar but not in polar environments. Recent studies suggest that the distinction between static and dynamics IMHBs is a pivotal concept to design new oral drugs in the bRo5 chemical space.[^5^, ^14^] In fact, dynamic but not static IMHBs may contribute to a molecular chameleon able to adapt its properties to the environment and thus cross membranes. For instance, dynamic IMHBs are responsible for the unexpected high permeability of cyclosporin A.^[13]^

**Figure 5:**
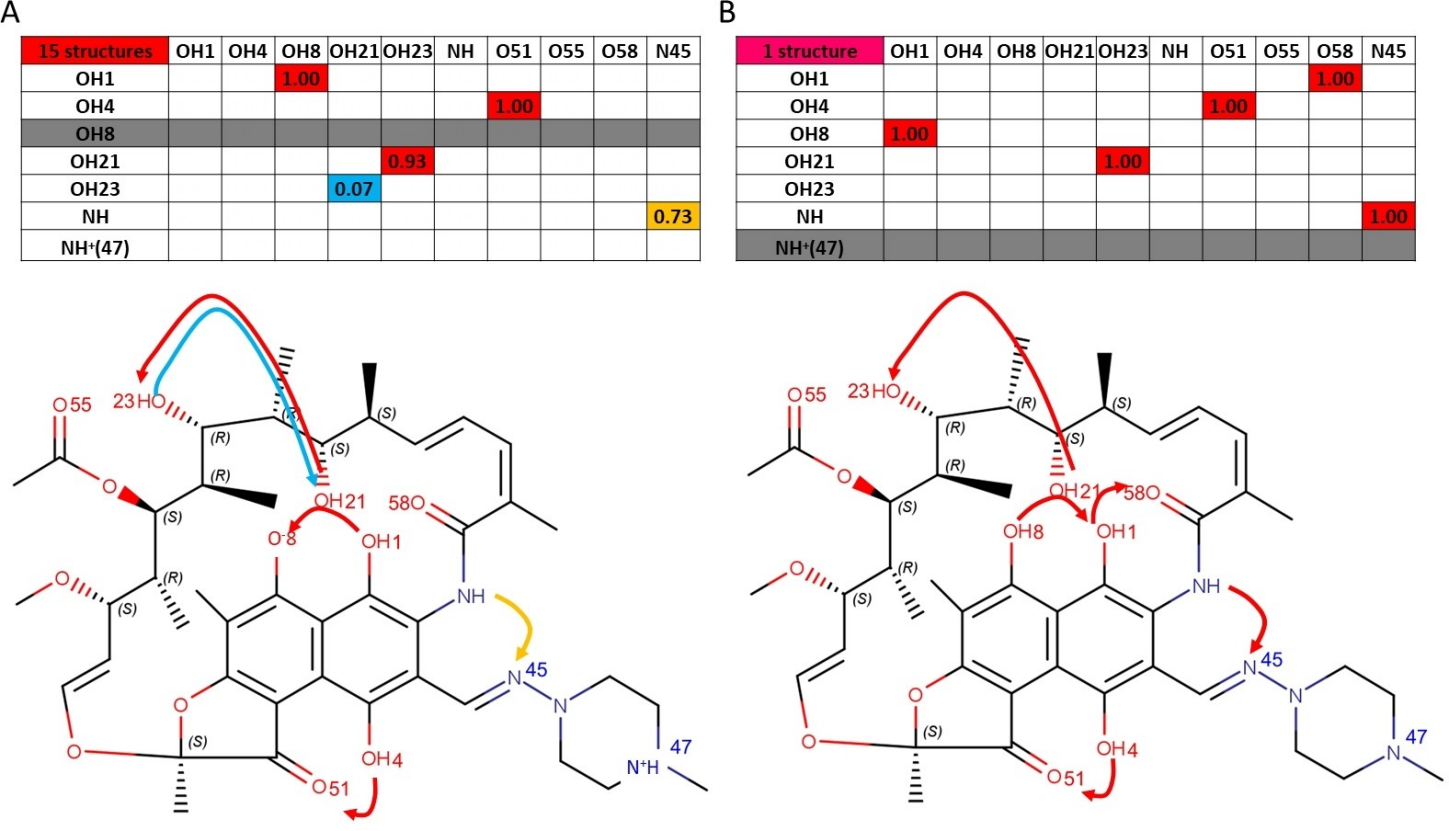
IMHB pattern deduced from crystal structures: A) zwitterionic form (15 structures); B) neutral form (1 structure). In red IMHB_frac_ values greater than 0.8, in yellow IMHB_frac_ values greater than 0.5 and less than 0.8 and, finally, in cyan IMHB_frac_ values lower than 0.5.

The 16 CSD structures were carefully inspected with Maestro to analyze IMHB patterns (see Methods). Graphical and numerical results are in Figure 5. The fraction of molecules showing IMHB with respect to the total number of structures (IMHB_frac_) was calculated for each IMHB. For example, the IMHB OH1‐OH8 is present in all the structures, thus a value of IMHB_frac_ equal to 1 is reported.

A network of IMHBs is present in the naphthohydroquinone system of both the zwitterionic (Figure 5A) and the neutral form (Figure 5B). The most relevant differences between the two species concern a) the hydroxyl OH1 that in the neutral form maximizes the number of IMHBs by acting both as an acceptor and a donor group (Figure 5B) and b) the IMHB between the amidic nitrogen and the iminomethyl group that it is not always present in the zwitterionic species (Figure 5A). All the other IMHBs are formed in both species, for example, OH21‐OH23. Notably, all IMHBs observed were predicted with a high propensity of formation in Figure 2.

Overall rifampicin shows a considerable IMHB network mostly due to the naphthohydroquinone system and largely independent of the ionization state and thus of the crystallization environment.

### NMR experiments and determination of the conformational ensembles in solution

The solution conformational ensembles of rifampicin were obtained by NMR spectroscopy and analyzed with non‐standard algorithms.[^9^, ^10^, ^11^] A previous study^[14]^ highlighted the conformational flexibility of rifampicin generating an ensemble of conformations compatible with the NMR data using the NAMFIS algorithm.^[8]^ Although widely adopted and recognized as the standard method, NAMFIS consists in a reweighting procedure of a force‐field based conformational sampling; consequently, its results depend strongly on how good the force field is and how exhaustive the a priori sampling is (e. g., if a relevant conformation is not recorded in the sampling, it cannot be reweighted). In this study we prefer to adopt a different method in which the iterative approach we followed does not suffer from these limitations, because the force field is progressively changed to weight more heavily conformations that are compatible with the experimental data. Moreover, since it is built within the framework of the principle of maximum entropy, the iterative algorithm ensures that the solution is unique and that it is affected by the least subjective bias, minimizing the amount of hypotheses in the construction of the model.^[20]^

NMR experiments were performed both in CDCl_3_ and in D_2_O pH=5, to study the neutral and zwitterionic form of rifampicin. In order to confirm the presence of zwitterion and to evaluate the deprotonation site in weakly acidic D2O, the ^13^C resonances were also evaluated and compared in the two solvents (cf. the numeration of atoms in Figure 1). A deshielding of the C8 resonance and a shielding of C11 resonance are observed (Figure S3) allowing to identify the phenol group at C8 as the most acidic site which undergoes deprotonation in water. Shielding of carbonyl C11 resonance evidences its involvement in delocalizing the negative charge through the aromatic ring. In addition, it is possible to observe (from the comparison of ^1^H spectra in the two solvents) evidence of deshielding in the resonances of piperazine side chain methylene (H39, H40, H41, H42) and methyl (H38) groups as a consequence of the protonation of N47 in the zwitterion.

**Figure 6:**
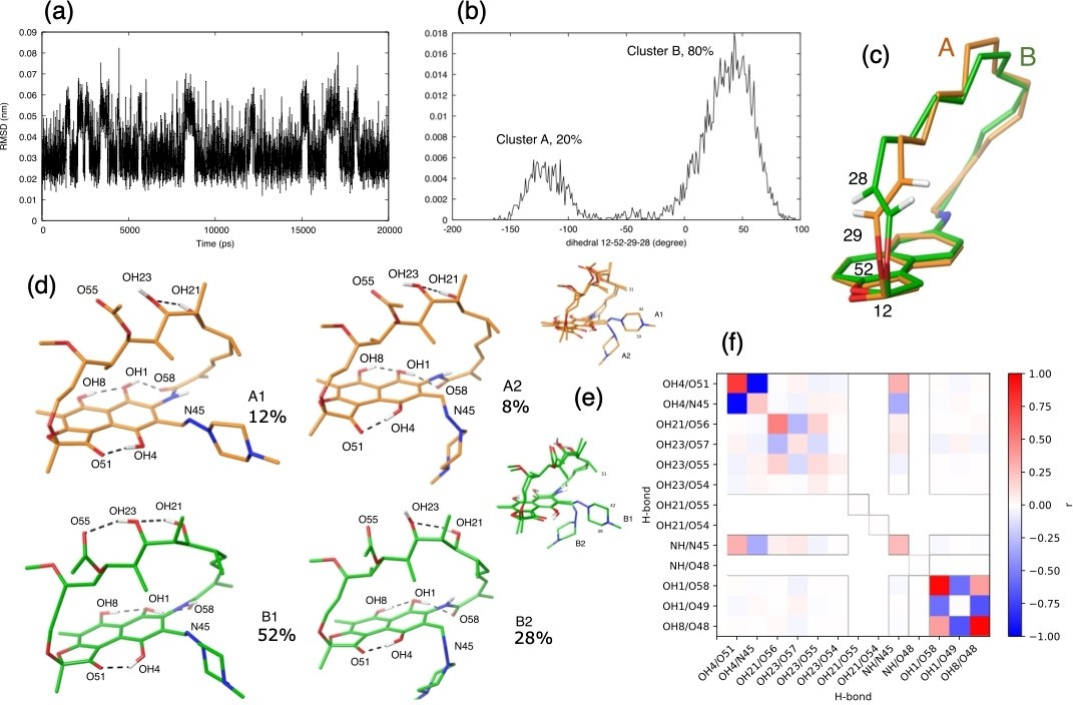
The results of the simulation of neutral rifampicin in chloroform. (a) The time course of the RMSD between the simulated molecule and the minimum‐energy structure indicate conformational changes. (b) The distribution of the dihedral between atoms 12‐52‐29‐28 of the macrocycle, calculated from the simulation. (c) The two peaks in the distribution define two clusters of conformations with different orientations of the macrocycle, labelled as A and B, (d) A clustering analysis of the simulated conformation reveals that clusters A and B can be further partitioned into sub‐clusters with different patterns of hydrogen bonds. (e) The comparison between sub‐clusters A1 and A2 and sub‐clusters B1 and B2 highlights a different orientation of the piperazine ring. (f) The formation probability of hydrogen bonds along the diagonal and their Pearson's correlation coefficients off‐diagonal; all pairs of hydrogen bonds that are able to form are considered.

From NOESY spectrum of rifampicin in CDCl_3_ a total of 28 NOEs are integrated and used for the further analysis, while only 7 NOEs are found in D_2_O. Applying the iterative simulation algorithm to neutral rifampicin (spectra in CDCl_3_), correcting the force field to reproduce the NOE data in chloroform, leads to a decrease of χ^2^ between the simulated and the experimental NOE intensities from 44.6 to 1.73 (Figures S4 and S5). The simulation obtained from the corrected force field displays conformational fluctuations on the RMSD scale of 0.07 nm, that is not negligible for such a small molecule (Figure 6a). In particular, the dihedrals that define the orientation of the groups 28CH and 29CH displays a bimodal distribution (Figure 6b), corresponding to two main clusters of conformations that the macrocycle can adopt, labelled as A and B in Figure 6c, with an equilibrium probability of 20 % and 80 %, respectively. The different values of this dihedral result in two equilibrium distances between group 34CH3 and OH4 (see free‐energy profile of Figure S6) which oscillates between 0.25 in cluster A and 0.4 nm in cluster B and is compatible with the observed NOESY crosspeak (112‐86/88 in Figure S5). A cluster analysis of the simulated conformations reveals a finer partitioning into four subclusters, characterized by a different orientation of the piperazine ring (Figure 6e). Cluster B1 is the most populated, with a probability of 52 %.

Only three hydrogen bonds of the naphthohydroquinone core (H4−O51, H1−O58, H8−O48) are stable in most conformations; the others fluctuate and display different correlation with each other (Figure 6f and Table S2). For example, NH−N45 and H4−O51 are rather correlated, while H4−N45 and H4−O51 are anticorrelated. Hydrogen bond NH−N45 characterizes clusters A2 and B2, in which the (4‐methyl‐1‐piperazinyl)‐iminomethyl is oriented under the plane of the naphthohydroquinone ring. Hydrogen bonds involving the aliphatic hydroxy groups, in particular OH21−O56, are more likely in cluster A. The formation of the other IMHBs seems uncorrelated on the clustering of the molecule.

**Figure 7:**
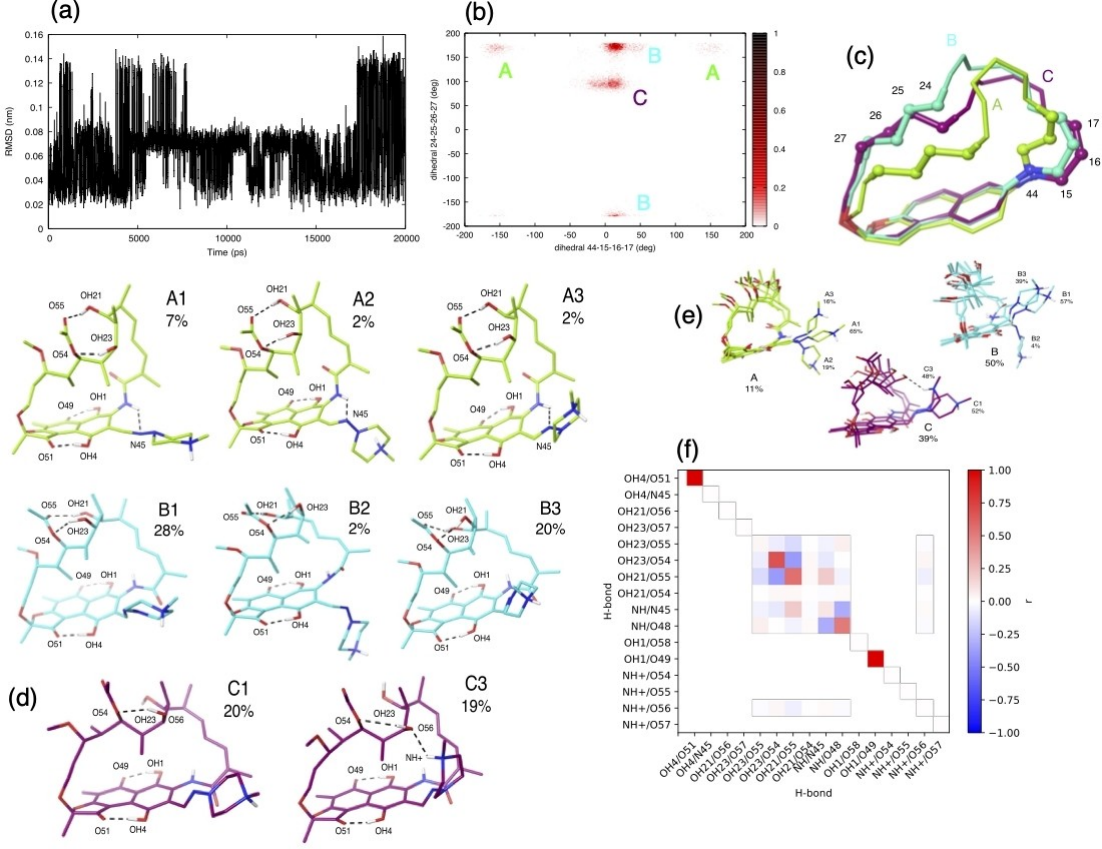
The results of the simulation of zwitterionic rifampicin in D2O. (a) The time course of the RMSD to the crystallographic structure shows fluctuations between at least three conformations. (b) The distribution associated with two dihedrals of the macrocycles of the three clusters. (d) The result of the clustering analysis. (e) The comparison between sub‐clusters. (f) the formation probability (on the diagonal) and the correlation (off‐diagonal) between hydrogen bonds; all pairs of hydrogen bonds that are able to form are considered.

The application of the iterative algorithm to zwitterionic rifampicin to simulate its behavior in water causes the χ^2^ between the simulated and the experimental NOE intensities to decrease from 20.3 to 0.28 (Figure S7 and S8). The conformational variability in water seems to be even larger than that in chloroform, with conformational changes on the scale of 0.14 nm (Figure 7a). The macrocycle, as characterized by its dihedrals, displays three main clusters (Figure 7b and the free energy profile of Figure S9), labelled as A, B and C in Figure 7c.

Different clusters display different propensities of formation of hydrogen bonds, but with patterns that are less strict than in chloroform. There are correlations in the formation of hydrogen bonds as well, but its degree is overall weaker than in chloroform (Figure 7f and Table S3 in the SUPPORTING INFORMATION); in particular, in water there are not pairs which are strongly mutually exclusive.

The stable hydrogen bonds of the naphthohydroquinone core are similar to those in chloroform, with some differences due to the deprotonation of group OH8 and protonation of N47 in water. As the negative charge is delocalized through the aromatic ring from O49 to O51 (as verified by NMR experiments), hydrogen bonds involving these groups are stronger than for the neutral rifampicin, and it is observed from very high probability of formation of interactions OH4−O51, OH1−O49.

OH21 interacts with O55 with high probability in clusters A and B but not in cluster C in which is mainly exposed to solvent. OH23 can interact with O54 with high probability in all conformations, but in cluster C becomes predominant. Amide NH interactions are also cluster‐dependent, in fact based on dihedral angle 44‐15‐16‐17, it is oriented toward N45 in cluster A, whereas in clusters B and C is mainly oriented toward O48. A RMSD‐based clustering of the simulated conformations suggests a finer partitioning into 7 subclusters, with different orientation of the piperazine ring (Figure 7d). At variance with the case in chloroform, in water there is a dominant orientation of the piperazine ring, which is in plane of the naphthohydroquinone core in all clusters.

Moreover, it is possible to recognize a third orientation of the side chain, probably due to its different ionization state in water, more folded towards the macrocyclic ring, in which the overall structure assumes a more spherical shape.

### Comparison of crystallographic and NMR structures

**Figure 8:**
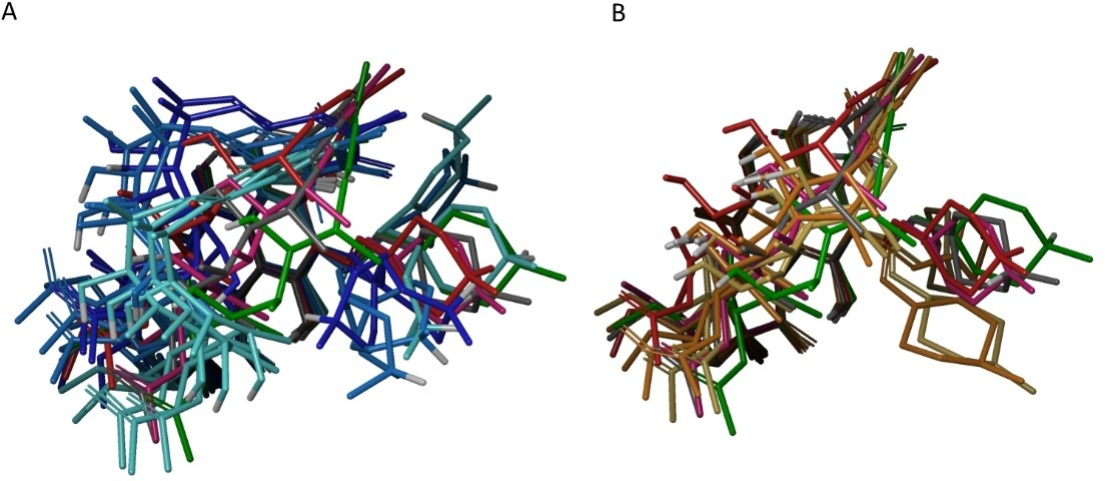
Superposition of NMR spectroscopy with the crystallographic relevant structures, one for each cluster. Crystallographic structures are colored according to Table 2 whereas different nuances of blue and yellow are used for NMR structure obtained in water and in chloroform, respectively. A) View perpendicular to the naphthohydroquinone moiety; B) lateral view.

The analysis of the experimental structures and of the IMHB network reported above reveals that conformations observed in solution and in solid states are different. In particular, the crystallographic structures are not observed in the NMR solutions (Figure 8). This is due to the higher degree of flexibility shown by the ansa substructure in solution than in the solid state.

**Figure 9:**
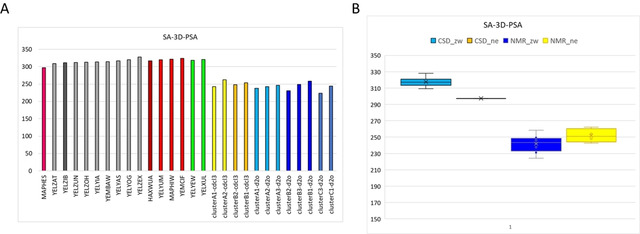
SA‐3D‐PSA values calculated on CSD and NMR conformations. A) Bars due to the crystallographic structures are colored on the basis of CSD clusters (Table 2) whereas yellow and orange colors were used for NMR conformations present in chloroform and water, respectively. B) Box plot of SA‐3D‐PSA values calculated on CSD (in cyan and in orange the zwitterionic and the neutral form respectively) and on NMR conformations (in blue and in yellow the zwitterionic and the neutral form respectively, different nuances of blue and yellow are used to distinguish the different clusters).

To get more insight into the impact of these conformational variations on the molecular property profile of rifampicin, a polarity descriptor, the polar molecular surface area (SA‐3D‐PSA) was calculated for all the NMR and crystal structures (Figure 9A).

The SA‐3D‐PSA of the unique neutral rifampicin structure present in the CSD, MAPHES, is 297.33 Å^2^, whereas the corresponding values for the zwitterionic form range from 309.33 Å^2^, YELZAT, to 324.06 Å^2^, YEMCIF. These values suggest that neutral rifampicin is slightly less polar than the zwitterionic species in the solid state (Figure 9B). This is in line with the presence of an additional IMHB. Moreover, the SA‐3D‐PSA range is about 14 Å^2^ and confirms that small structural variations in the crystallographic structures have a small impact on molecular properties. For instance, Rossi Sebastiano and coworkers showed that in the presence of intramolecular interactions, a SA‐3D‐PSA difference between 60 and 80 A^2^ is present in a set of some bRo5 drugs.^[5]^

The SA‐3D‐PSA values calculated on the NMR structures, range from 242.67 Å^2^ to 262.37 Å^2^ for the neutral rifampicin in chloroform and from 224.07 Å^2^ to 262.37 Å^2^ for the zwitterionic form in water. The polarity range is slightly smaller in chloroform (about 20 Å^2^) than in water (about 38 Å^2^) and suggests that in water rifampicin shows a greater flexibility than in chloroform. According to these data, the polarity of the two species is very close (Figure 9B).

The difference in the SA‐3D‐PSA calculated in polar and nonpolar media has been considered an index of chameleonicity.^[14]^ In chameleonic compounds, this difference is positive because these molecules mask their polar moieties in apolar environments and thus reduce the exposed polar surface. In the solid state, we assume that the neutral form of rifampicin is present when the crystallization process has been performed in a nonpolar and aprotic environment; the reverse is true for the zwitterion. In NMR experiments, chloroform is the nonpolar media populated by neutral rifampicin, whereas in water the zwitterionic species are more populated. The box plot in Figure 9B suggests that rifampicin has modest chameleonic properties both in the solid state and in solution. This lack of chameleonicity of rifampicin is in line with the results reported in a recent paper by Kihlberg and co‐workers.^[14]^ In this study the authors explored the NMR solution conformational behavior of rifampicin (and of other three drugs) at pH=7.0 using the NAMFIS algorithm. At this pH rifampicin is present both as zwitterion (76 %) and as anion (24 %) (Figure 1C). The different experimental conditions may therefore explain the slightly different 3D‐PSA values (about 220 Å^2^) found in the NMR Swedish study in the two solvents. Remarkably, also this study confirmed that 3D‐PSA values are smaller in solution than in the solid state.

Generally speaking, cell permeability is favored by the presence of both static and dynamic IMHBs. In the case of rifampicin, crystallographic data emphasize the presence of static IMHBs (=present in both polar and nonpolar environment) in particular, in the naphthohydroquinone ring. Static IMHB impact molecular permeability by masking polar groups but they do not contribute to chameleonicity. On the other hand, NMR results suggest the presence of dynamic IMHBs (=present in nonpolar but not in polar environments), for instance the IMHB between the donor OH4 and the acceptor N45. These IMHBs are expected to promote chameleonicity which in turn favors membrane permeation. Notably, in rifampicin the presence of dynamic IMHBs does not produce a decrease in the SA‐3D‐PSA (Figure 9B) suggesting that their presence is not sufficient to promote a chameleonic behavior. In principle, chameleonicity may also be related to variations in the hydrophobic surface area (HySA). Therefore, we also calculated and compared the HySA for the X‐Ray and the NMR structures (Figures S18 and S19). Again, no relevant differences were observed. Therefore, we can hypothesize that rifampicin permeability is due to the presence of static IMHBs whereas the presence of dynamic IMHBs appears to be negligible in this respect.

## Conclusion

Experimental 3D structures are an information source of high relevance for large and flexible compounds with pharmacological potential. bRo5 molecules may in fact show complex IMHB patterns dependent on the environment, with potential impact on permeability and thus bioavailability. The availability of conformers population in different media should therefore be considered a preferential tool to be used in drug discovery to monitor properties variation in polar and nonpolar regions of membranes.

Crystallography is the most common supplier of experimental 3D structures, but it is suitable to catch stable interactions like static IMHBs whereas it shows significant limitation in the assessment of dynamic IMHBs. On the contrary, NMR is a valuable tool to assign the disposition of the molecule to form dynamic IMHBs. However, the interpretation of NMR spectra in terms of conformational ensembles is far from trivial. In the present work we employed a method that guarantees an exhaustive search of the heterogeneous set of conformations that, all together, give rise to the NMR spectrum.

In this study, we applied both methods to unravel the structural features driving rifampicin permeability. Our results suggest that the disposition of rifampicin to form dynamic IMHBs is not sufficient to determine a chameleonic behavior and that rifampicin permeability could be due to the presence of static IMHBs although other factors for rifampicin‘s capacity to permeate membranes remain to be further explored. These are important results in terms of designing new bRo5 drugs oral available.

## Methods

### Molecular descriptors calculation

Mol2 files used to calculate molecular descriptors used in the paper were obtained retrieving the SMILES code in the DrugBank (www.drugbank.com) and then converting such codes with CORINA (https://www.mn‐am.com/online_demos/corina_demo).

The Kier flexibility index (PHI), and the number of hydrogen bond donor (HBD) and acceptor (HBA) atoms, limited to nitrogen and oxygen atoms file, were calculated submitting mol2 files in DRAGON (version 7.0.10, 2017, https://chm.kode‐solutions.net).

Furthermore, mol2 files were used to evaluate HB propensity using the propensity calculation tool present in Mercury^[7]^ (https://www.ccdc.cam.ac.uk/). This tool predicts which donors and acceptors form hydrogen bonds in a crystal structure, based on the statistical analysis of hydrogen bonds in the Cambridge Structural Database.^[16]^

### X‐ray dataset

All instances of crystal structure data for rifampicin were extracted from the CSD (www.ccdc.cam.ac.uk) searching by common name, rifampicin.

All entries were downloaded and edited to delete solvent molecules and spots due to unresolved peaks.

Finally, data were loaded in Maestro and the structures were checked for the correct atom valency and bond order.

The representation of the molecules with thermal ellipsoids was obtained using Mercury with default parameters.

### Structure alignment

Firstly, structures were aligned using the maximum common structure (MCS) algorithm implemented in the RDKit (www.rdkit.com). The MCS is a wide used algorithm for generating a one‐to‐one atom correspondence between two molecules when the atom sequence is not the same in the two structures. From these alignments the RMSD value was determined and used to drive the partition of structures in clusters.

Finally, all structures were aligned with Chimera (ver. 1.15, UCSF Chimera Home Page) using the naphthohydroquinone moiety as template in order to obtain a new alignment that allowed an easy representation of the different clusters.

### IMHB pattern determination

The IMHB were determined using standard tools available in the educational version of Maestro (ver. 2020‐1, www.schrodinger.com). The Maestro criteria were selected and checked using Mercury, in particular an HB is formed when the distance between the hydrogen and the acceptor atom is less than 2.8 Å, the value of the angle formed by donor, hydrogen and acceptor atoms is at least 120° and the angle between hydrogen, acceptor and the atom bound to the acceptor is greater than 90° (www.schrodinger.com).

The same criteria were used to study the results of MD simulations.

IMHB were manually annotated.

### HB propensity calculation

The propensity to form hydrogen bonds is evaluated through a statistical analysis of information obtained from the crystallographic structures collected in the Cambridge Structural Database (CSD). The main steps of the procedure are briefly listed below. First, the hydrogen bond donor and acceptor atoms and HBs in which they are involved are identified in the crystal structures. In a second step, the existence of a HB between potential and acceptor atoms is verified on the basis of geometrical criteria and recorded as a two‐state variable (true or false). Some descriptive properties are also collected in this step, such as which functional group the donor/acceptor atoms belong to, e. g. hydroxyl, carboxylic, etc. These data are then used as both qualitative and quantitative parameters to develop a two‐state probability model based on information extracted from CSD. Finally, the model allows the computation of a knowledge‐based probability index, the propensity, for the formation of a certain HB in the molecule between a pair of acceptor/donor atoms.

### NMR experiments

The two ionization states of rifampicin were characterized by one‐ and two‐dimensional NMR experiments, using 2 mM solutions in CDCl_3_ and 0.4 mM D_2_O (pH 5), for the neutral and the zwitterion forms respectively. The lower concentration in D_2_O was chosen in order to avoid aggregation of rifampicin at higher concentrations.

Assignments of ^1^H and ^13^C chemical shifts of the two states of the molecule were made through 1H‐1H (COSY, TOCSY, NOESY) and ^1^H‐^13^C correlation experiments (HSQC, HMBC) (Figures S10–S15) and are reported in Tables S4 and S5 of Supp. Mat. All spectra were acquired using Bruker Avance III 400 MHz and Avance 600 MHz instruments operating a 300 K. Where necessary, water suppression was carried out by excitation sculpting pulse sequence. Evaluation of the chemical shift of specific ^1^H and ^13^C allowed to verify the intramolecular proton transfer in aqueous environment and to identify the involved groups (Table S6).

For the conformational analysis three independent replicas of NOESY spectra (with 48 scans and 256 increments) were collected in both solvents, using a 600 MHz spectrometer at 300 K, with a mixing time of 400 ms (Figures S5 and S6). Intensity of the NOESY cross‐peaks was measured as the maximum height of the peak and averaged over the three replicated spectra, in order to assign to each cross peaks a standard error (Tables S7 and S8). To avoid the presence of spin diffusion, the build‐up curves were plotted using NOE cross peaks obtained at 200, 400 and 700 ms. The mixing time of 400 ms was due to the absence of spin diffusion (Figure S16).

### Determination of conformational ensembles

The simulations are performed with the algorithm described in refs.[^9^, ^10^, ^11^] to correct force fields to match experimental NOEs. Atom and bond types are assigned using the Antechamber tool of Amber18 program and charges are obtained with AM1‐BCC method. The starting potential is the GAFF force field^[22]^ in implicit solvent. Simulations are carried out with Gromacs 4.5.5, controlled by a tailor‐made code responsible for the correction of the potential and the calculation of the NOEs. For the simulations in chloroform, we used the neutral structure sketched in Figure 1a in vacuum with a relative dielectric constant of the implicit solvent of 4.8. For the simulations in water, we used the zwitterionic structure of Figure 1b in implicit GBSA water.

In both cases, a total of 100 iterations are carried out at temperature of 300 K; each iteration lasted for 20 ns with a time step of 2 fs. A set of 5000 conformations are recorded at each iterative step to calculate the NOEs.

As discussed in ref. [9], we used an implicit‐solvent model because in explicit solvent the reweighting scheme is poorly effective, the change in energies being much smaller than the total energy of the (rifampicin+water) system and because it is more difficult to equilibrate it (cf. also Figure S17).

### Polar and hydrophobic surface area calculation

Three‐dimensional PSA (named SA‐3D‐PSA) was calculated following the protocol described in Rossi Sebastiano et al.^[5]^ Briefly, the SA‐3D‐PSA was calculated in PyMOL v1.7.0.1 using molecular surface areas calculated from atomic van der Waal's radii or solvent‐accessible surface areas calculated using a solvent molecule radius of 1.4 Å. Atoms were assigned as “polar” either based solely on atom type (O, N, and attached H) or by also including atoms with absolute partial charges from the PM3 semiempirical method above a defined threshold. Partial charges are calculated submitting structures derived from CSD and NMR in Spartan ′18 (ver. 1.4.4, www.wavefunction.com).

HySA was calculated using Maestro with default parameters.

## Conflict of interest

The authors declare no conflict of interest.

## Supporting information

As a service to our authors and readers, this journal provides supporting information supplied by the authors. Such materials are peer reviewed and may be re‐organized for online delivery, but are not copy‐edited or typeset. Technical support issues arising from supporting information (other than missing files) should be addressed to the authors.

Supplementary
