# Supplementary material for: Managing Experimental 3D Structures in the Beyond‐Rule‐of‐5 Chemical Space: The Case of Rifampicin
Source: Chemistry. 2021 Jun 10;27(40):10394–404. doi: 10.1002/chem.202100961 (PMC8361677; doi:10.1002/chem.202100961)

Chemistry–A European Journal

Supporting Information

Managing Experimental 3D Structures in the Beyond-Rule-of-5 Chemical Space: The Case of Rifampicin

Giuseppe Ermondi+, Francesca Lavore+, Maura Vallaro, Guido Tiana, Francesca Vasile,* and Giulia Caron*

Supporting Information

Tables

Table S1. Result of the search of rifampicin in CSD with information about the solvent used to obtain the crystal, the temperature of the experiment, the ionization state of rifampicin, the publication year and the DOI of the paper where the structure was published for the first time (all information extracted form CSD files)

Table S2: Distribution of hydrogen bonds along the conformations is chloroform. Probability of each interaction is showed for all conformations, for clusters A and B and for each subcluster A1, A2, B1, B2. H, D and A represents id numbers of hydrogen, donator ad acceptor atoms involved in the interaction.

Table S3: Distribution of hydrogen bonds along the conformations is water. Probability of each interaction is showed for all conformations and for clusters A, B and C. H, D and A represents id numbers of hydrogen, donator and acceptor atoms involved in the interaction.

Table S4: NMR assignment of neutral rifampicin in CDCl3.

Table S5: NMR assignment of zwitterion rifampicin in D2O.

Table S6: Chemical shift difference between groups undergoing ionization in D2O.

Table S7: The average and standard deviation over three replicate experiments of the NOE intensities for neutral rifampicin. Equivalent spins are grouped.

Table S8: The average and standard deviation over three replicate experiments of the NOE intensities for zwitterionic rifampicin. Equivalent spins are grouped.

Figures

Figure S1. Structure of representative structures of three clusters with thermal ellipsoids. A) HAXWUA; B) MAPHES; C) YELZOH

Figure S2: The neutral (a) and zwitterionic (b) form of rifampicin. In parenthesis the numbers associated with Oxygen and Hydrogens, respectively, used throughout this paper.

Figure S3: The superposition of HMBC spectra of Rifampicin, in CDCl3 (red) and D2O (blu), displays the resonance variation of C8 and C11 in the two solvents. At the left is reported a representative resonance structure in which the molecule assumes a quinonic structure that explains deshielding of C8 and shielding of C11 in the zwitterionic form.

Figure S4: The χ^2 between the simulated and the experimental NOE intensities as a function of the number of iterations of the correction algorithm for rifampicin in chloroform.

Figure S5: The experimental NOE intensities for neutral rifampicin in chloroform (green bars) compared with those obtained from the current model after optimization of the energies (red bars) and from MD simulations with the GAFF force field (blue bars). The error bars in the experimental data are estimated as the standard deviation of the triplicate experiment. The error bars in the simulations indicate the fluctuations around the average.

Figure S6: The free energy of neutral rifampicin in chloroform displays two minima corresponding to the two main clusters.

Figure S7: The χ^2 between the simulated and the experimental NOE intensities as a function of the number of iterations of the correction algorithm for zwitterionic rifampicin in D_2O.

Figure S8: The experimental NOE intensities for zwitterionic rifampicin in D_2O (green bars) compared with those obtained from the current model after optimization of the energies (red bars) and from MD simulations with the GAFF force field (blue bars). The error bars in the experimental data are estimated as the standard deviation of the triplicate experiment. The error bars in the simulations indicate the fluctuations around the average.

Figure S9: The free energy of zwitterionic rifampicin in water display three minima that correspond to the three clusters displayed in Fig. 3.

Figure S10: ^1H NMR spectrum of Rifampicin in $CDCl_3$.

Figure S11: ^1H NMR spectrum of Rifampicin in D_2O at pH 5.

Figure S12: ^1H-^{13}C-HSQC spectrum of Rifampicin in $CDCl_3$.

Figure S13: ^1H-^{13}C-HSQC spectrum of Rifampicin in D_2O at pH 5.

Figure S14: expansion of ^1H-^1H-NOESY spectra of Rifampicin in $CDCl_3$ (mixing time=400 ms).

Figure S15: expansion of ^1H-^1H-NOESY spectra of Rifampicin in D_2O (mixing time=400 ms).

Figure S16. Build-up curve for theNOE crosspeak between proton 17 and 30 both $CDCl_3$ (purple) and D_2O (green).

Figure S17: A comparison between the experimental NOEs in D_2O and those obtained from a MD simulation in explicit water with the GAFF force field. The associated χ^2 is 28.3.

Figure S18. SA-HyPSA values calculated on all the NMR conformations (in blue and in yellow the zwitterionic in water and the neutral form in chloroform respectively). Box and whistle plot for A) the entire structures; B) the naphtohydroquinone system fused with a furanone ring (moiety A); C) the ansa (moiety B) and, D) the (4-methyl-1-piperazinyl)-iminomethyl chain (moiety C).

Figure S19. SA-HyPSA values calculated on all the X-Ray conformations (in blue and in yellow the zwitterionic and the neutral form respectively). Box and whistle plot for A) the entire structures; B) the naphtohydroquinone system fused with a furanone ring (moiety A); C) the ansa (moiety B) and, D) the (4-methyl-1-piperazinyl)-iminomethyl chain (moiety C).

Table S1. Result of the search of rifampicin in CSD with information about the solvent used to obtain the crystal, the temperature of the experiment, the ionization state of rifampicin, the publication year and the DOI of the paper where the structure was published for the first time (all information extracted form CSD files)

Refcode	R-factor (R%)	Solvation	T (K)	Ionization state	Pub. Year	DOI
HAXWUA	4.82	pentahydrate	130	zw	2012	10.1107/S0108270112015296
LOPZEX*	2.64	anydrous	298	ne	2014	10.1039/C4CE01157K
LOPZEX01*	6.02	anydrous	100	ne	2018	10.1016/j.molstruc.2017.10.083
MAPHES	5.38	1,1,1-trichloroethane solvate	130	ne	2012	10.1039/c2ob00008c
MAPHIW	4.00	methanol solvate trihydrate	130	zw	2012	10.1039/C2OB00008C
OWELOS***	9.04	ethylene glycol solvate dihydrate	173	ne***	2011	10.1021/mp100459y
OWELUY***	13.67	ethylene glycol solvate dihydrate	100	ne***	2011	10.1021/mp100459y
RIFAMP**	5.94	pentahydrate	295	ne**	1975	10.1107/S0567740875005407
YELXUL	8.41	1-pentanol solvate hydrate	130	zw	2018	10.1021/acs.cgd.7b01121
YELYAS	7.91	2-pentanol dichloromethane solvate hemihydrate	130	zw	2018	10.1021/acs.cgd.7b01121
YELYEW	8.42	hexan-1-ol solvate monohydrate	130	zw	2018	10.1021/acs.cgd.7b01121
YELYIA	4.35	propan-2-ol solvate monohydrate	120	zw	2018	10.1021/acs.cgd.7b01121
YELYOG	7.28	pentan-3-ol solvate	130	zw	2018	10.1021/acs.cgd.7b01121
YELYUM	4.33	ethanol solvate hydrate	130	zw	2018	10.1021/acs.cgd.7b01121
YELZAT	6.50	acetone solvate hydrate	130	zw	2018	10.1021/acs.cgd.7b01121
YELZEX	7.64	butan-2-one solvate	120	zw	2018	10.1021/acs.cgd.7b01121
YELZIB	2.69	acetone solvate monohydrate	130	zw	2018	10.1021/acs.cgd.7b01121
YELZOH	6.03	butan-1-ol solvate hemihydrate	120	zw	2018	10.1021/acs.cgd.7b01121
YELZUN	6.22	propan-1-ol solvate monohydrate	120	zw	2018	10.1021/acs.cgd.7b01121
YEMBAW	7.48	2-butanol solvate hemihydrate	120	zw	2018	10.1021/acs.cgd.7b01121
YEMCIF	9.18	dimethyl sulfoxide solvate hydrate	130	zw	2018	10.1021/acs.cgd.7b01121

* Powder technique
** Wrong ionization state
*** R values greater than 8 and ionization state uncertain due to the presence of water and protic solvents

Table S2: Distribution of hydrogen bonds along the conformations in chloroform. Probability of each interaction is showed for all conformations, for clusters A and B and for each subcluster A1, A2, B1, B2. H, D and A represents id numbers of hydrogen, donator ad acceptor atoms involved in the interaction.

HB type	%All conf.	%A	%B	%clA1	%clA2	%clB1	%clB2	H	D	A
OH4/O51	76,9	76,9	76,9	76,7	77,2	73,0	84,2	112	59	51
OH4/N45	22,0	22,0	22,1	23,1	20,7	26,0	14,8	112	59	45
OH21/O56	49,3	59,8	49,7	59,4	60,3	49,6	50,0	111	57	56
OH23/O57	11,2	16,9	11,1	18,3	15,3	10,8	11,6	110	56	57
OH23/O55	13,8	24,2	13,3	23,3	25,3	13,4	13,2	110	56	55
OH23/O54	0,1	0,3	0,1	0,2	0,5	0,1	0,1	110	56	54
OH21/O55	0,0	0,0	0,0	0,0	0,0	0,0	0,0	111	57	55
OH21/O54	0,0	0,0	0,0	0,0	0,0	0,0	0,0	111	57	54
NH/N45	27,3	21,9	28,6	13,7	31,8	21,4	42,0	107	44	45
NH/O48	0,0	0,0	0,0	0,0	0,0	0,0	0,0	107	44	48
OH1/O58	96,3	96,7	96,2	97,2	96,0	96,1	96,5	108	48	58
OH1/O49	1,2	0,9	1,3	0,9	0,9	1,5	1,0	108	48	49
OH8/O48	97,5	98,2	97,0	98,8	97,5	97,0	97,0	109	49	48

Table S3: Distribution of hydrogen bonds along the conformations in water. Probability of each interaction is showed for all conformations and for clusters A, B and C. H, D and A represents id numbers of hydrogen, donator and acceptor atoms involved in the interaction.

HB type	% All conf.	%clust. A	%clust. B	%clust.C	H	D	A
OH4/O51	100	100	100	100	111	59	51
OH4/N45	0,0	0,0	0,0	0,0	111	59	45
OH21/O56	0,0	0,0	0,0	0,0	110	57	56
OH23/O57	0,0	0,0	0,0	0,0	109	56	57
OH23/O55	3,5	1,3	6,6	0,2	109	56	55
OH23/O54	67,4	51,6	50,2	94,0	109	56	54
OH21/O55	55,3	95,5	88,1	2,0	110	57	55
OH21/O54	0,12	0,00	0,80	0,21	110	57	54
NH/N45	8,6	71,0	0,3	1,7	107	44	45
NH/O48	51,4	8,9	59,7	52,8	107	44	48
OH1/O58	0,0	0,0	0,0	0,0	108	48	58
OH1/O49	100	100	100	100	108	48	49
NH+/O54	0,0	0,0	0,0	0,0	117	47	54
NH+/O55	0,0	0,0	0,0	0,0	117	47	55
NH+/O56	0,3	0,0	0,0	0,8	117	47	56
NH+/O57	0,0	0,0	0,0	0,0	117	47	57

Table S4: NMR assignment of neutral rifampicin in CDCl3.

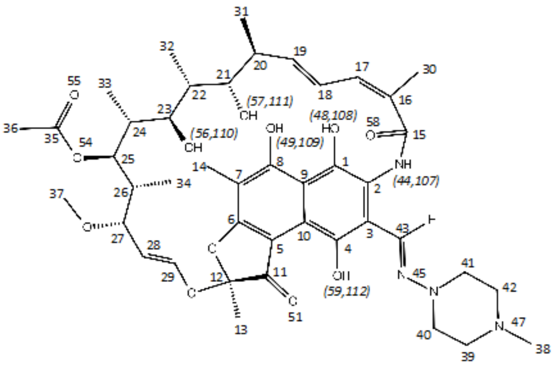

Atom	^{13}C (ppm)	^{1}H (ppm)	Number of protons, multiplicity, J (Hz)
1	138,6	-	-
2	120,3	-	-
3	109,3	-	-
4	149,0	-	-
5	104,5	-	-
6	174,5	-	-
7	107,1	-	-
8	168,8	-	-
9	112,8	-	-
10	118,3	-	-
11	195,6	-	-
12	108,6	-	-
13	21,4	1,82	3H, s
14	7,5	2,25	3H, s
15	169,3	-	-
16	129,4	-	-
17	135,2	6,41	1H, d, $^{3}J_{(17-18)}$ 11,2
18	123,2	6,60	1H, ddd, $^{3}J_{(18-19)}$15,7 ; $^{3}J_{(18-17)}$ 11,2 ; $^{4}J_{(18-20)}$ 1,1
19	142,8	5,95	1H, dd, $^{3}J_{(19-18)}$ 15,7 ; $^{3}J_{(19-20)}$ 4,90
20	38,6	2,40	1H, m
21	70,7	3,79	1H, d, $^{3}J_{(21-20)}$ 9,80
22	33,5	1,74	1H, m
23	77,1	3,03	1H, m
24	37,5	1,55	1H, m
25	74,5	4,97	1H, d, $^{3}J_{(25-26)}$ 10,4
26	39,6	1,38	1H, m
27	76,7	3,50	1H, ddd, $^{3}J_{(27-28)}$ 6,7; $^{4}J_{(27-29)}$ 1,05 ; $^{3}J_{(27-26)}$ 2,12
28	118,6	5,13	1H, dd, $^{3}J_{(28-29)}$12,8 ; $^{3}J_{(28-27)}$ 6,7
29	142,6	6,23	1H, dd, $^{3}J_{(29-28)}$12,8 ; $^{4}J_{(29-27)}$ 1,05
30	20,7	2,10	3H, s
31	17,8	0,90	3H, d, $^{3}J_{(31-20)}$ 7,05
32	10,9	1,04	3H, d, $^{3}J_{(32-22)}$ 7,05
33	8,5	0,62	3H, d, $^{3}J_{(33-24)}$ 6,90
34	8,9	-0,27	3H, d, $^{3}J_{(34-26)}$ 6,93
35	172,2	-	-

36	20,7	2,08	3H, s
37	57,0	3,07	3H, s
38	46,0	2,35	3H, s
40/41	50,3	3,15	4H, m
39/42	54,1	2,57	4H, m
43	134,5	8,31	1H, s
OH21	-	3,45	1H, s
OH23	-	3,63	1H, d, $^{3}J_{(OH23\text{-}23)}$ 4,89
OH4	-	12,03	1H, s
NH	-	13,22	1H, s
OH1/OH8	-	13,50	brs

Table S5: NMR assignment of zwitterion rifampicin in D2O.

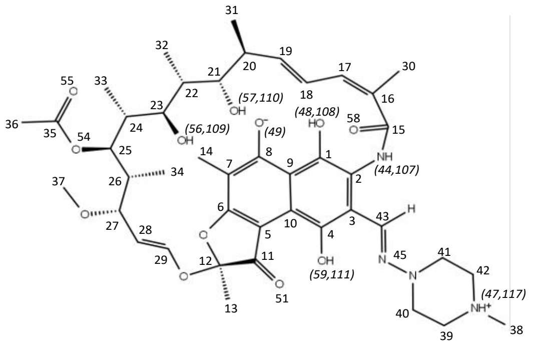

Atom	^{13}C (ppm)	^{1}H (ppm)	Number of protons, multiplicity, J (Hz)
1	-	-	-
2	117,8	-	-
3	114,6	-	-
4	145,0	-	-
5	-	-	-
6	173,5	-	-
7	104,8	-	-
8	184,0	-	-
9	-	-	-
10	-	-	-
11	187,3	-	-
12	109,8	-	-
13	21,2	1,73	3H, s
14	6,5	1,96	3H, s
15	171,5	-	-
16	130,6	-	-
17	133,5	6,39	1H, d, $^{3}J_{(17-18)}$ 11,7
18	126,7	6,83	1H, dd, $^{3}J_{(18-19)}$ 16,7 ; $^{3}J_{(18-17)}$ 11,7
19	139,4	6,12	1H , dd, $^{3}J_{(19-18)}$ 16,7 ; $^{3}J_{(19-20)}$ 7,5
20	37,4	2,31	1H, m
21	74,3	3,74	1H, d, $^{3}J_{(21-20)}$ 11,5
22	32,5	1,78	1H, m
23	76,3	3,02	1H, dd, $^{3}J_{(23-24)}$ 10,93 ; $^{3}J_{(23-22)}$ 2,4
24	37,7	1,31	1H, m
25	74,5	5,00	d, $^{3}J_{(25-26)}$ 11,07
26	39,9	1,13	1H, m
27	77,4	3,38	1H, d, $^{3}J_{(27-28)}$ 8,67
28	118,4	5,05	dd, $^{3}J_{(28-29)}$ 12,9 ; $^{3}J_{(28-27)}$ 8,55
29	143,2	6,27	1H, d, $^{3}J_{(29-28)}$ 12,9
30	19,6	1,97	3H, s
31	17,4	0,86	3H, d, $^{3}J_{(31-20)}$ 7,3
32	9,5	0,88	3H, d, $^{3}J_{(32-22)}$ 7,44
33	7,9	0,53	3H, d, $^{3}J_{(33-24)}$ 7,2
34	8,1	-0,32	3H, d, $^{3}J_{(34-26)}$ 7,28
35	173,5	-	-
36	20,1	2,02	3H, s
37	55,9	2,96	3H, s

38	42,9	2,78	3H, s
39/40/41/42	52,0	3,16	brs
43	136,6	8,00	1H, s

Table S6: Chemical shift difference between groups undergoing ionization in D2O.

	C8 (ppm)	C11 (ppm)	C38 (ppm)	H38 (ppm)
$CDCl_3$	168,8	195,6	46	2,35
D_2O	184	187,3	42,9	2,78
Abs diff.	15,2	8,3	3,1	0,43

Table S7: The average and standard deviation over three replicate experiments of the NOE intensities for neutral rifampicin. Equivalent spins are grouped.

Atom i	Atom j	H_i (PDB id)	H_j (PDB id)	Average I_{ij}	σ_{ij}
OH4	34	112	86,87,88	1,18E+05	1,9E+04
OH4	33	112	83,84,85	4,99E+04	9,7E+03
43	33	106	83,84,85	1,09E+05	1,9E+04
18	31	114	77,78,79	7,80E+04	1,7E+04
18	21	114	67	1,33E+06	2,9E+04
17	30	113	74,75,76	9,55E+05	9,7E+03
29	34	117	86,87,88	1,98E+05	1,5E+04
29	37	117	92,93,94	2,06E+05	1,1E+04
29	27	117	73	6,11E+05	2,1E+04
19	31	115	77,78,79	6,85E+05	3,2E+04
19	20	115	66	6,86E+05	1,4E+04
28	34	116	86,87,88	6,60E+05	2,4E+04
25	34	71	86,87,88	7,75E+05	1,5E+04
25	33	71	83,84,85	9,34E+04	1,4E+04
28	26	116	72	3,07E+05	2,7E+04
25	26	71	72	1,72E+05	1,9E+04
28	27	116	73	4,46E+05	2,0E+04
25	23	71	69	3,20E+05	2,5E+04
21	33	67	83,84,85	6,17E+05	2,8E+04
21	31	67	77,78,79	4,85E+05	3,0E+04
21	24	67	70	1,42E+06	1,9E+04
27	34	73	86,87,88	1,37E+05	1,3E+04
23	33	69	83,84,85	4,71E+05	2,1E+04
23	32	69	80,81,82	6,13E+05	3,6E+04
22	33	68	83,84,85	1,36E+06	1,1E+05
24	34	70	86,87,88	1,41E+06	9,9E+04
26	34	72	86,87,88	8,56E+05	5,6E+03
26	33	72	83,84,85	8,57E+05	7,7E+04

Table S8: The average and standard deviation over three replicate experiments of the NOE intensities for zwitterionic rifampicin. Equivalent spins are grouped.

Atom i	Atom j	H_i (PDB id)	H_j (PDB id)	Average I_{ij}	σ_{ij}
18	21	113	67	2.43E+05	6.7E+04
17	30	112	74,75,76	2.78E+05	1.0E+05
21	24	67	70	5.49E+05	1.4E+05
27	26	73	72	2.91E+05	4.7E+04
26	34	72	86,87,88	3.28E+05	2.8E+03
23	22	69	68	4.35E+05	1.4E+04
23	24	69	70	2.36E+05	3.2E+04

Figure S1. Structure of representative structures of three clusters with thermal ellipsoids. A) HAXWUA; B) MAPHES; C) YELZOH

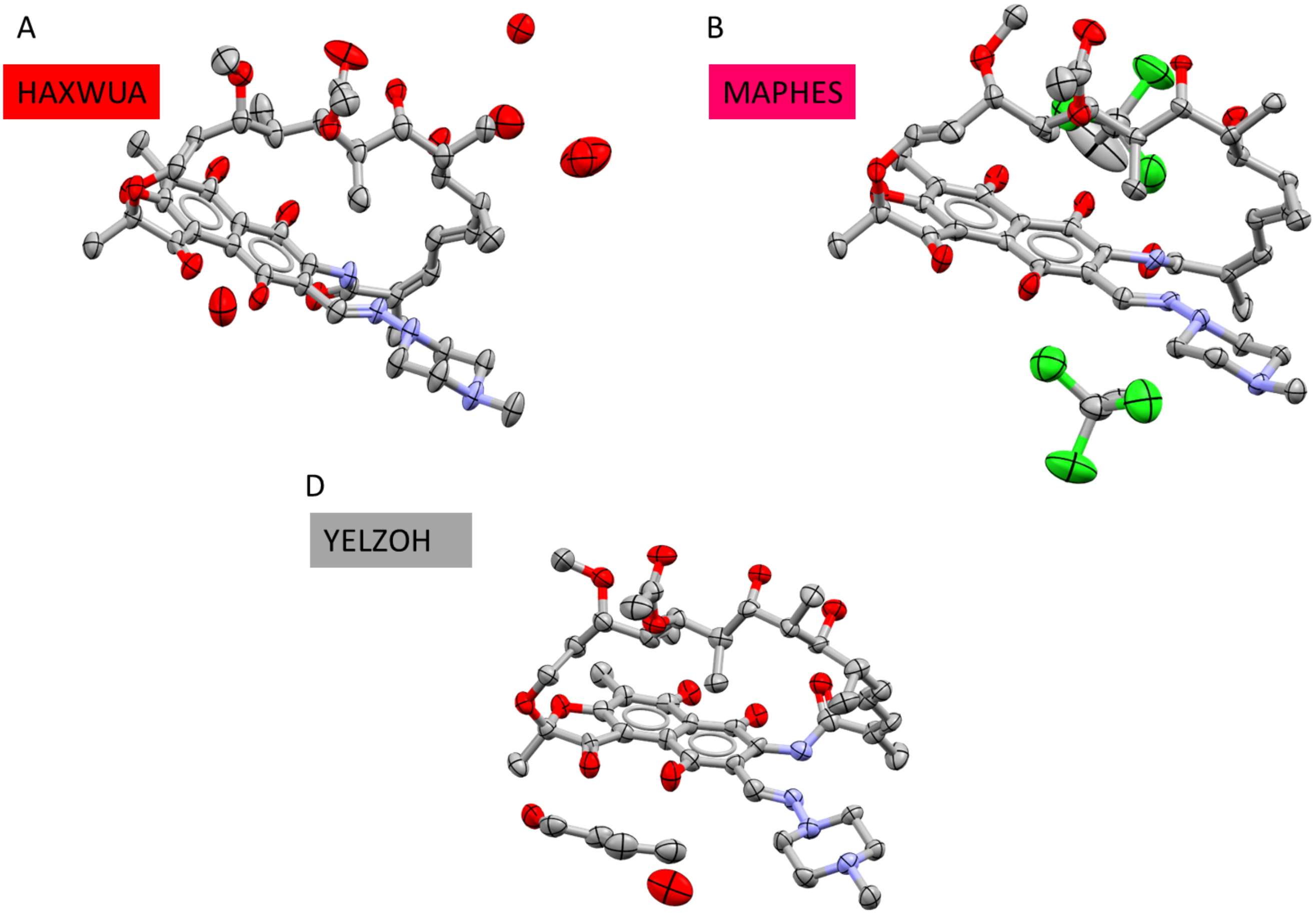

Figure S2: The neutral (a) and zwitterionic (b) form of rifampicin. In parenthesis the numbers associated with Oxygen and Hydrogens, respectively, used throughout this paper.

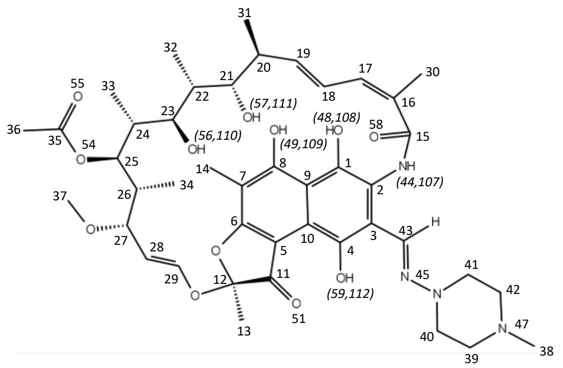

(a)

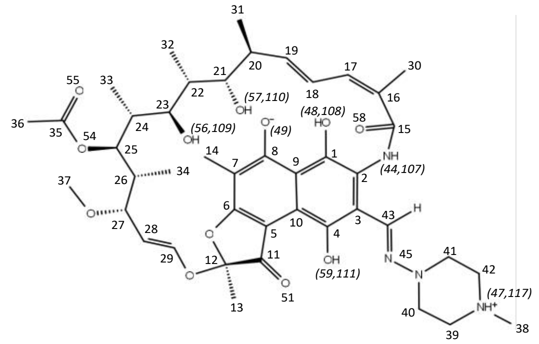

(b)

Figure S3: The superposition of HMBC spectra of Rifampicin, in CDCl3 (red) and D2O (blu), displays the resonance variation of C8 and C11 in the two solvents. At the left is reported a representative resonance structure in which the molecule assumes a quinonic structure that explains deshielding of C8 and shielding of C11 in the zwitterionic form.

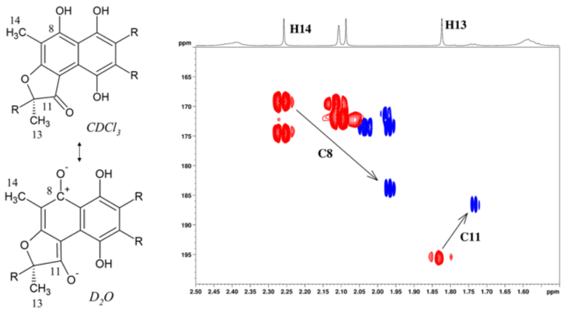

Figure S4: The χ^2 between the simulated and the experimental NOE intensities as a function of the number of iterations of the correction algorithm for rifampicin in chloroform.

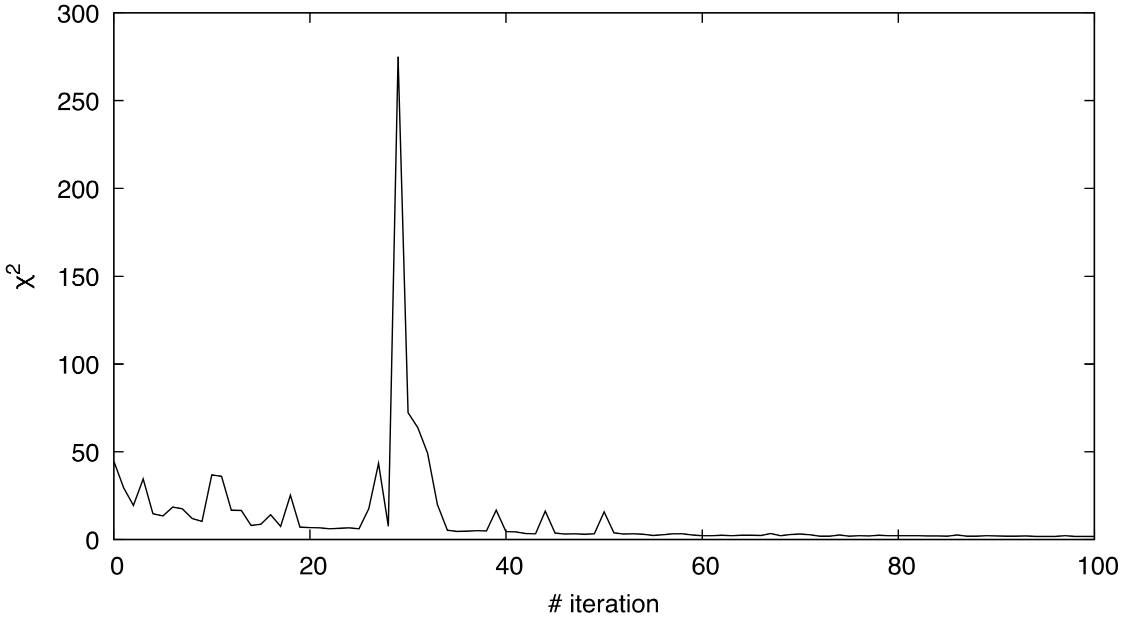

Figure S5: The experimental NOE intensities for neutral rifampicin in chloroform (green bars) compared with those obtained from the current model after optimization of the energies (red bars) and from MD simulations with the GAFF force field (blue bars). The error bars in the experimental data are estimated as the standard deviation of the triplicate experiment. The error bars in the simulations indicate the fluctuations around the average.

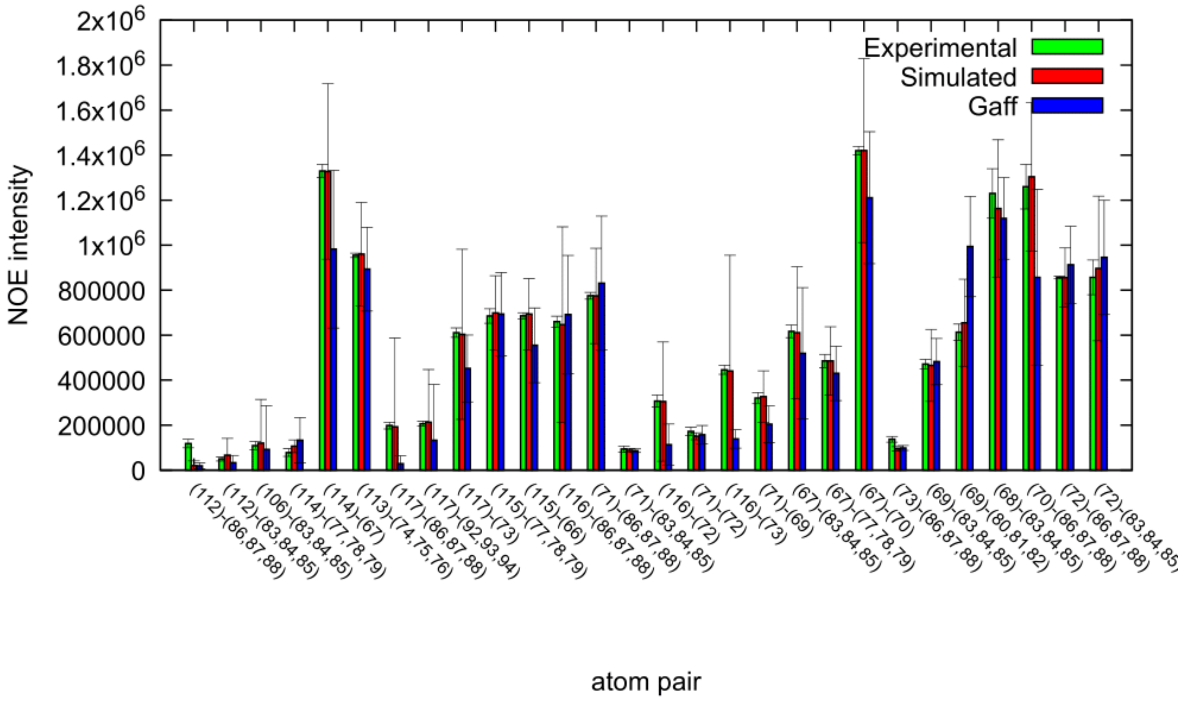

Figure S6: The free energy of neutral rifampicin in chloroform displays two minima corresponding to the two main clusters.

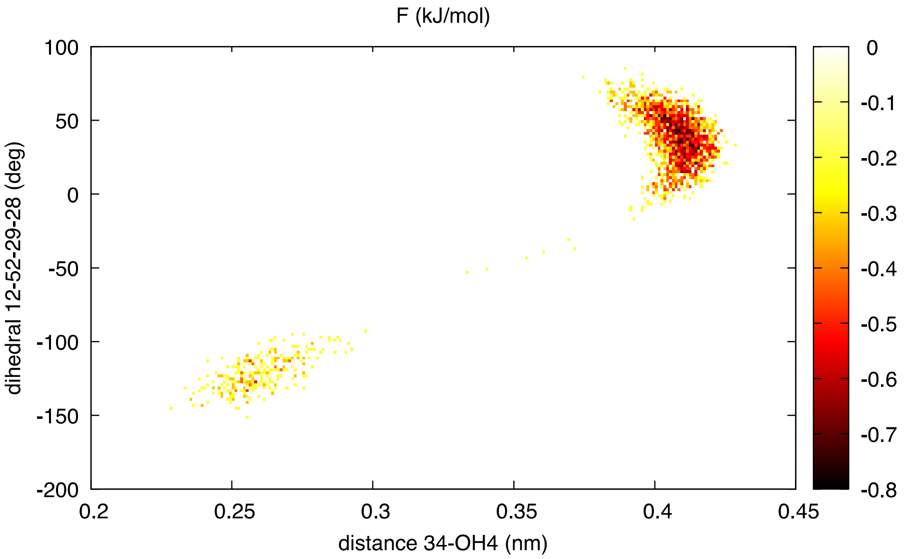

Figure S7: The χ^2 between the simulated and the experimental NOE intensities as a function of the number of iterations of the correction algorithm for zwitterionic rifampicin in D_2O.

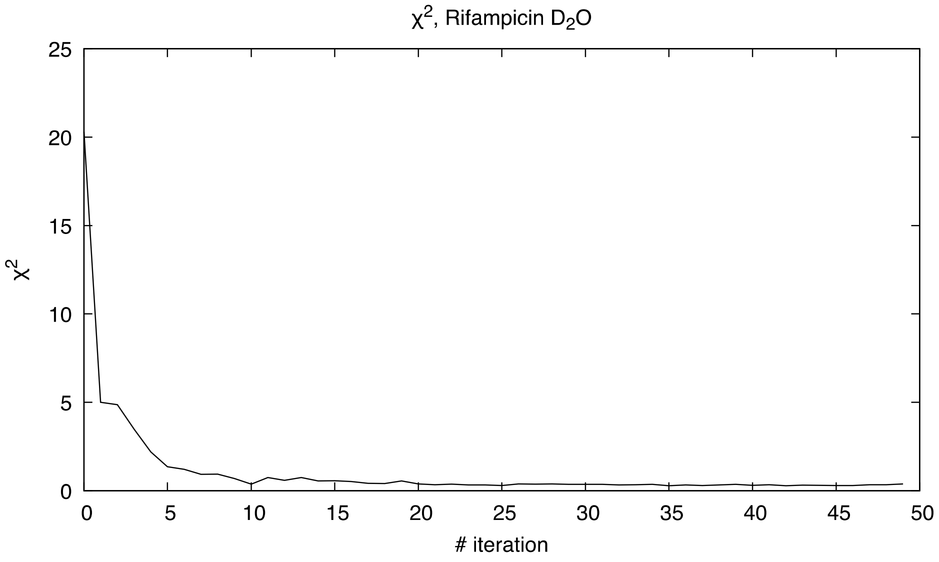

Figure S8: The experimental NOE intensities for zwitterionic rifampicin in D_2O (green bars) compared with those obtained from the current model after optimization of the energies (red bars) and from MD simulations with the GAFF force field (blue bars). The error bars in the experimental data are estimated as the standard deviation of the triplicate experiment. The error bars in the simulations indicate the fluctuations around the average.

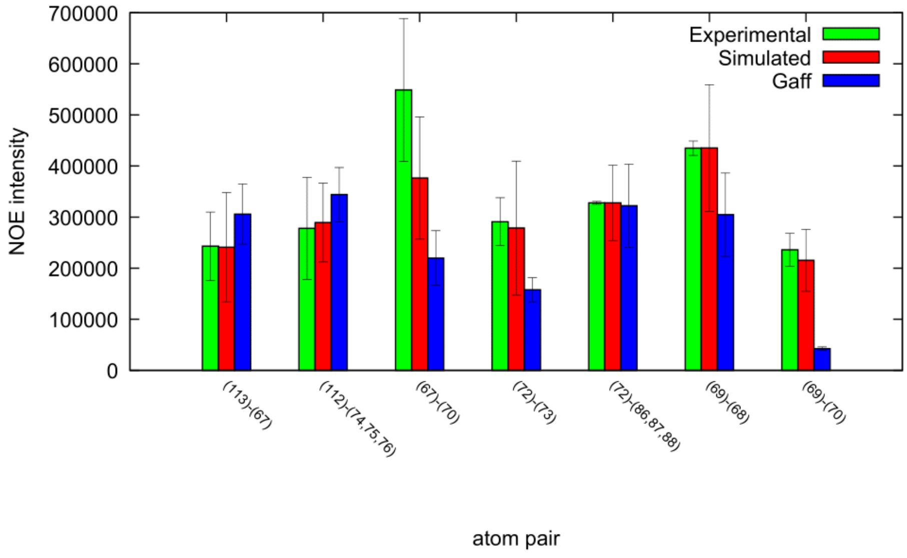

Figure S9: The free energy of zwitterionic rifampicin in water display three minima that correspond to the three clusters displayed in Fig. 3.

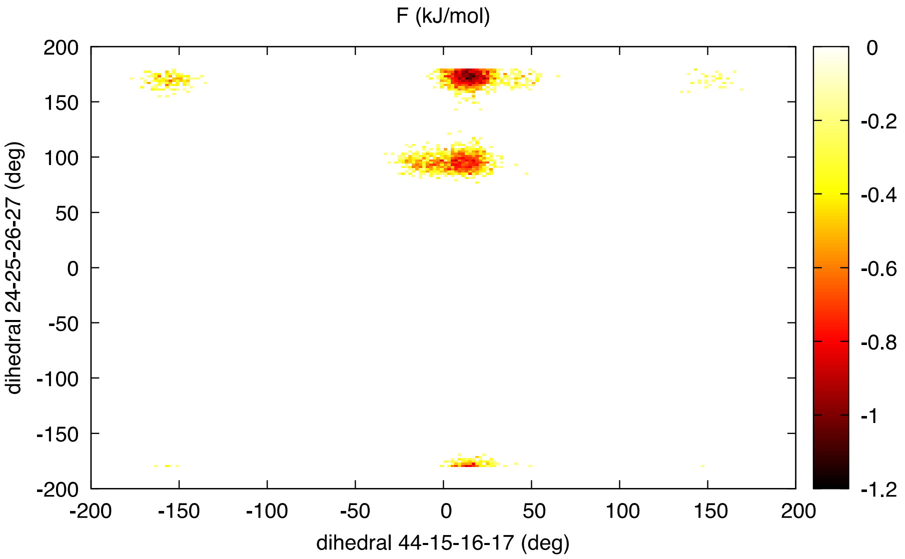

Figure S10: ^{1}H NMR spectrum of Rifampicin in $CDCl_3$.

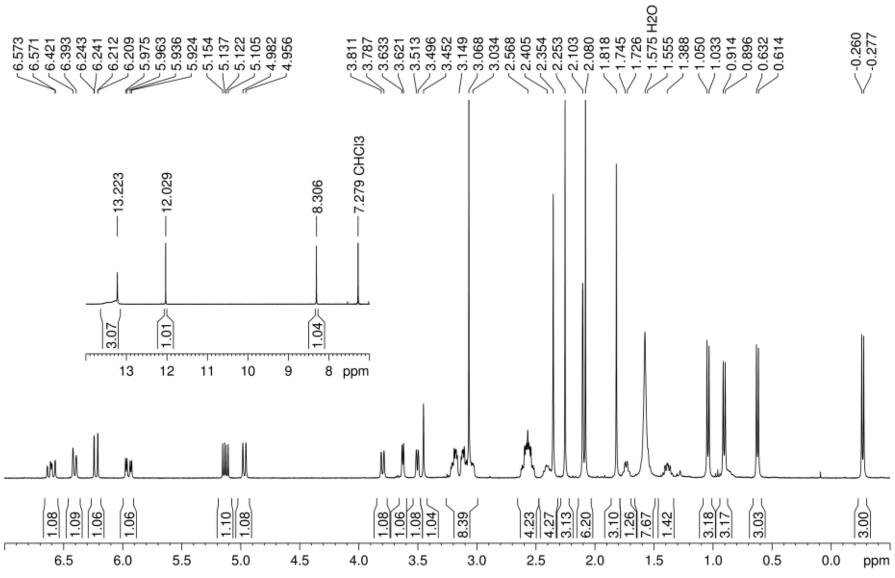

Figure S11: ^{1}H NMR spectrum of Rifampicin in D_2O at pH 5.

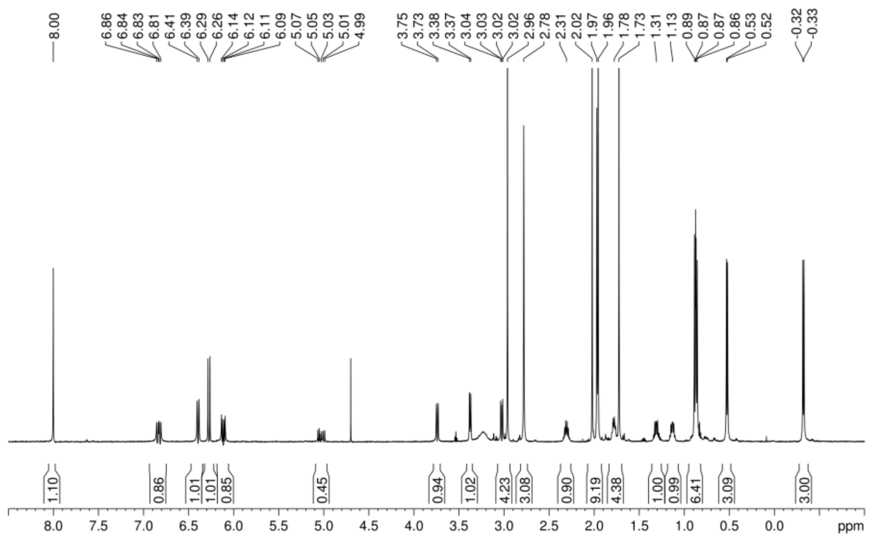

Figure S12: ^{1}H-^{13}C-HSQC spectrum of Rifampicin in $CDCl_3$.

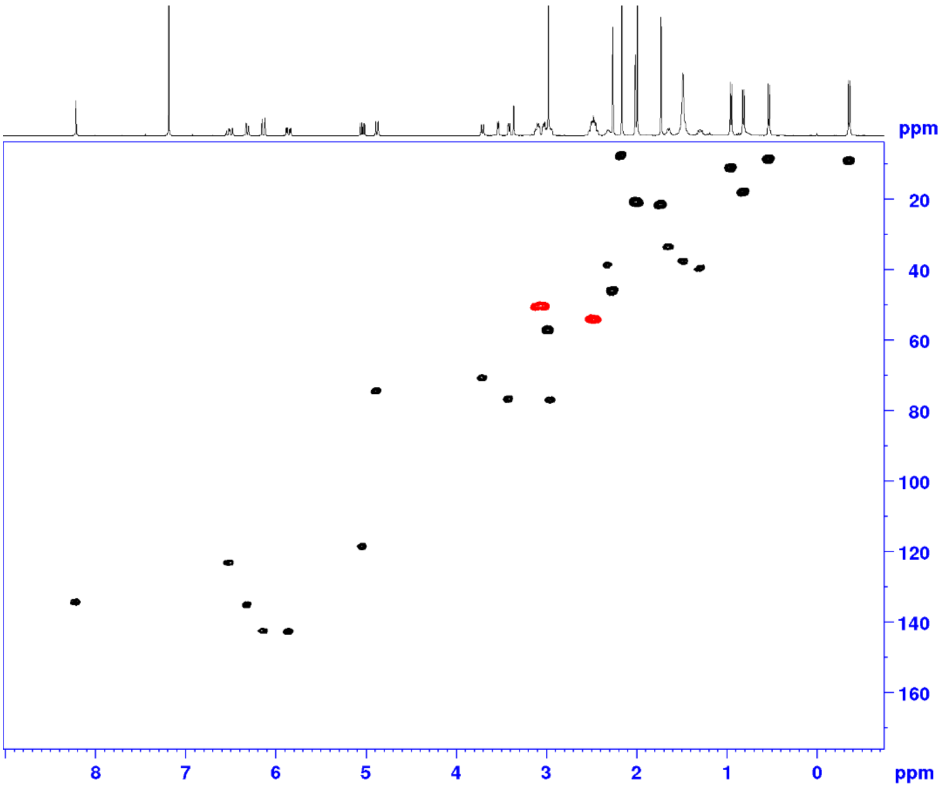

Figure S13: ^{1}H-^{13}C-HSQC spectrum of Rifampicin in D_2O at pH 5.

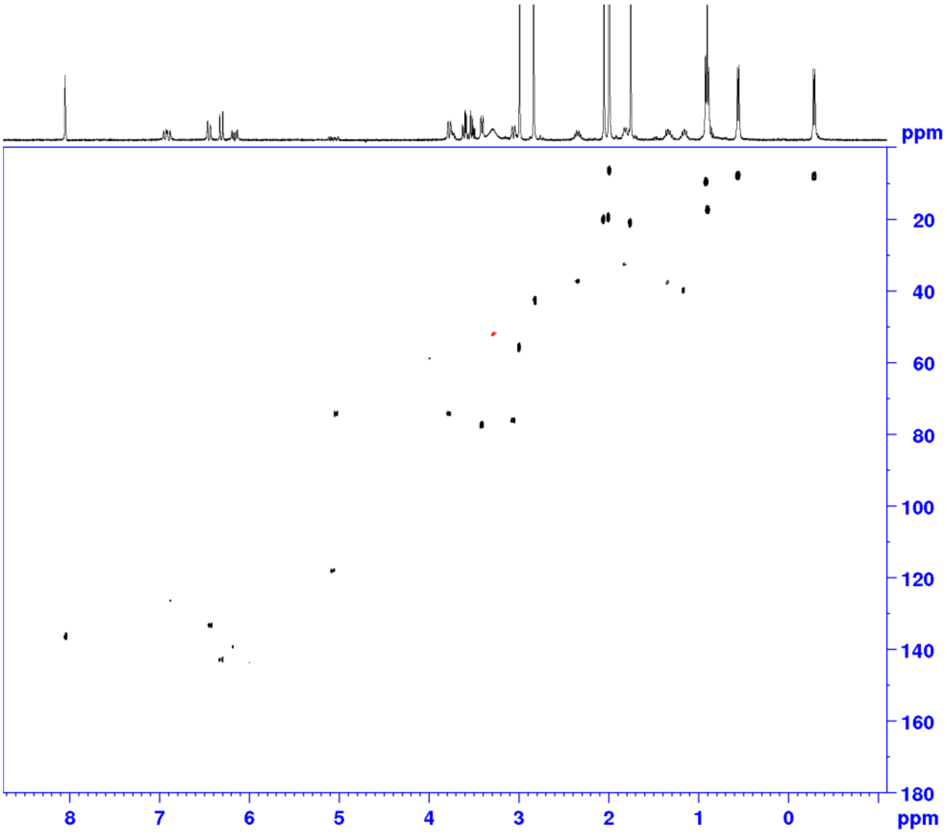

Figure S14: expansion of ^1H-^1H-NOESY spectra of Rifampicin in $CDCl_3$ (mixing time=400 ms).

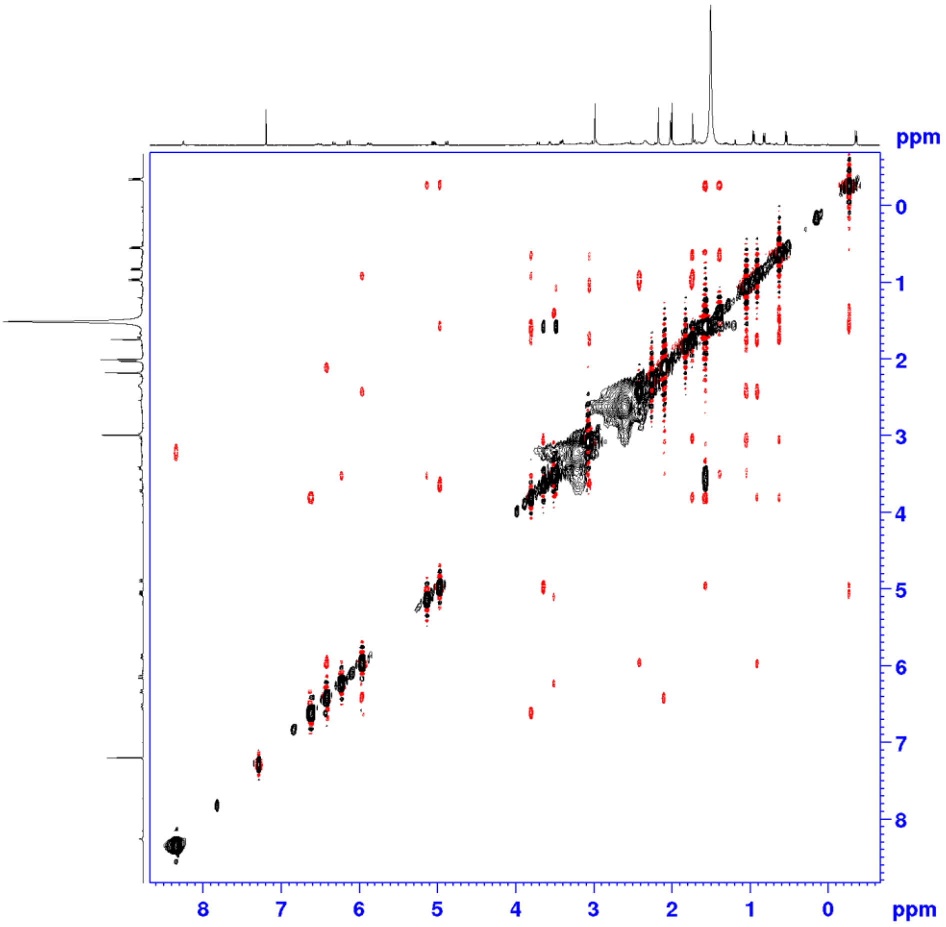

Figure S15: expansion of ^1H-^1H-NOESY spectra of Rifampicin in D_2O (mixing time=400 ms).

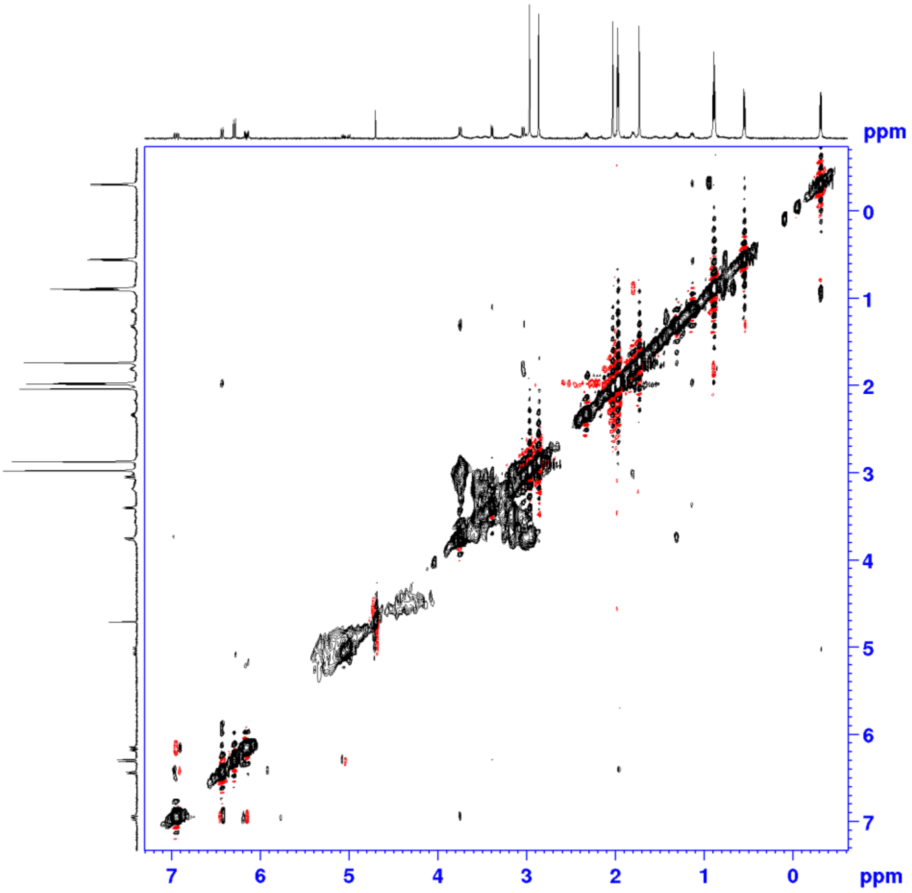

Figure S16. Build-up curve for theNOE crosspeak between proton 17 and 30 both $CDCl_3$ (purple) and D_2O (green).

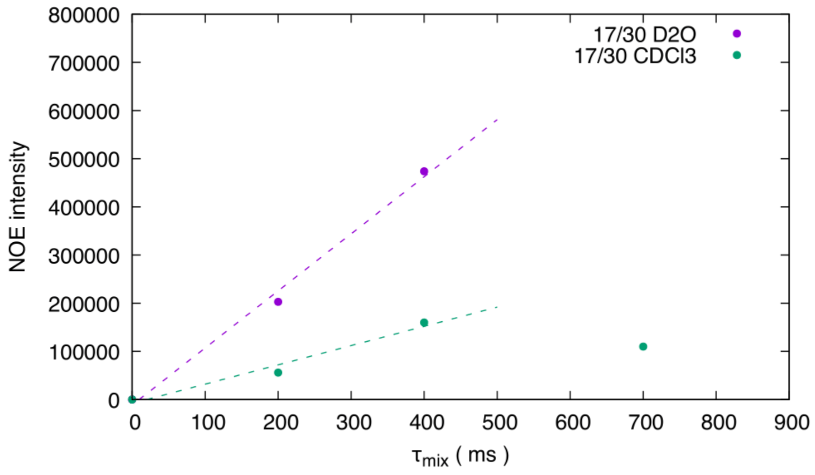

Figure S17: A comparison between the experimental NOEs in D_2O and those obtained from a MD simulation in explicit water with the GAFF force field. The associated χ^2 is 28.3.

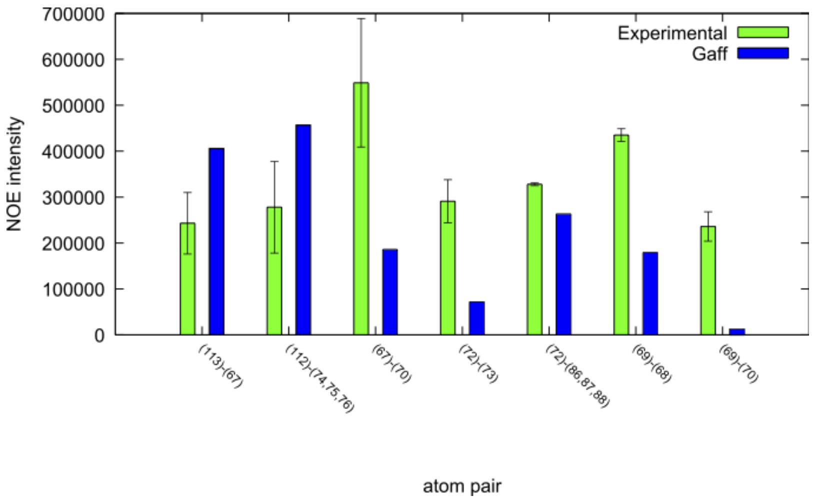

Figure S18. SA-HyPSA values calculated on all the NMR conformations (in blue and in yellow the zwitterionic in water and the neutral form in chloroform respectively). Box and whistle plot for A) the entire structures; B) the naphtohydroquinone system fused with a furanone ring (moiety A); C) the ansa (moiety B) and, D) the (4-methyl-1-piperazinyl)-iminomethyl chain (moiety C).

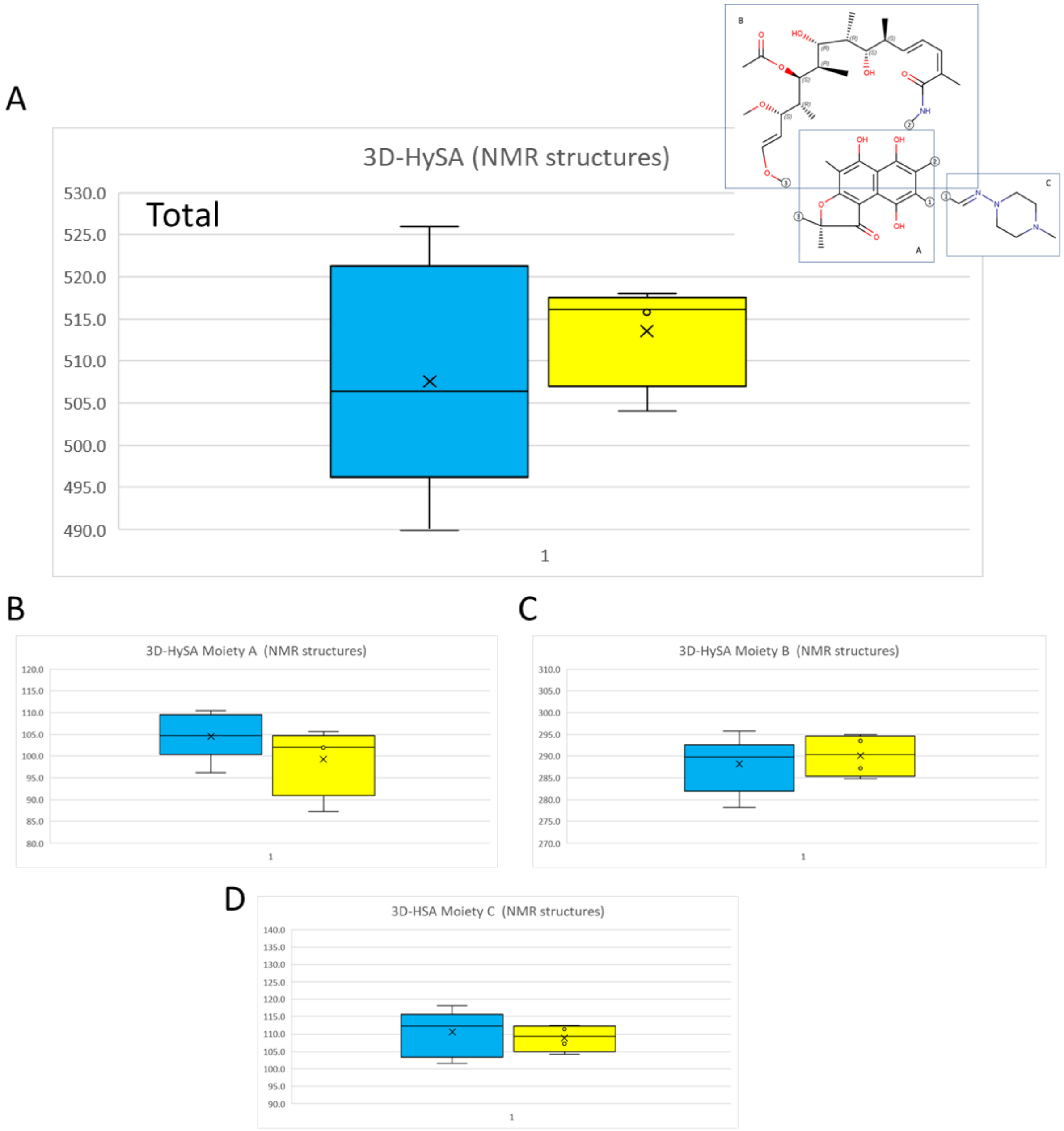

Figure S19. SA-HyPSA values calculated on all the X-Ray conformations (in blue and in yellow the zwitterionic and the neutral form respectively). Box and whistle plot for A) the entire structures; B) the naphtohydroquinone system fused with a furanone ring (moiety A); C) the ansa (moiety B) and, D) the (4-methyl-1-piperazinyl)-iminomethyl chain (moiety C).

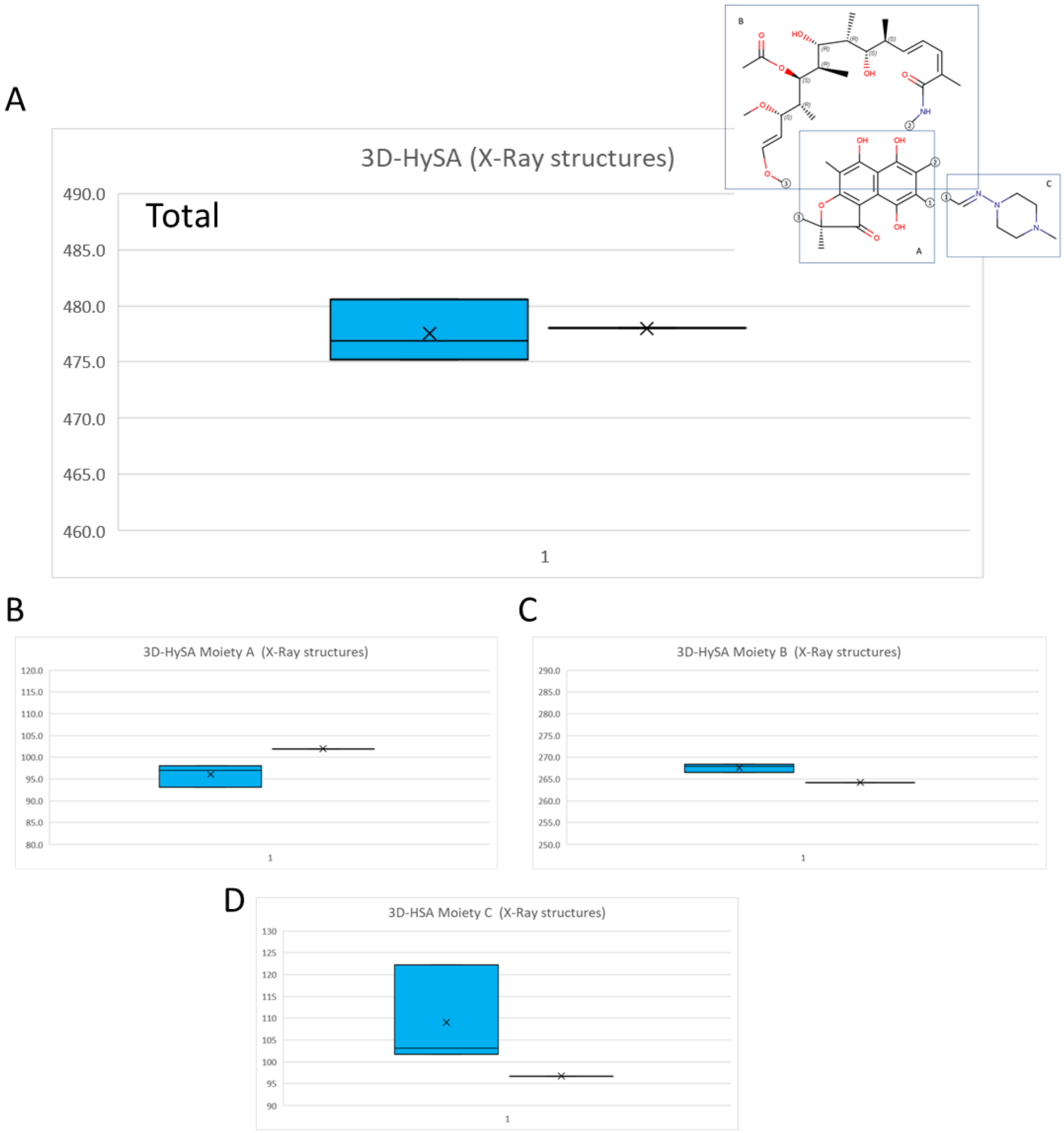

Supplement: Supplementary file 1 — Supplementary [file CHEM-27-10394-s001.pdf]
